# Carbon nanodots constructed by ginsenosides and their high inhibitory effect on neuroblastoma

**DOI:** 10.1186/s12951-023-02023-w

**Published:** 2023-07-28

**Authors:** Yingnan Jiang, Lizhi Xiao, Jifeng Wang, Tenghui Tian, Guancheng Liu, Yu Zhao, Jiajuan Guo, Wei Zhang, Jiawen Wang, Changbao Chen, Wenyi Gao, Bai Yang

**Affiliations:** 1 Jilin Ginseng Academy, Changchun University of Chinese Medicine, Changchun, 130117 P. R. China; 2 State Key Laboratory of Supramolecular Structure and Materials, College of Chemistry, Jilin University, Changchun, 130012 P. R. China

**Keywords:** Natural bioactive, Ginsenosides, Carbon nanodots, Nanomedicine, Inhibition of neuroblastoma

## Abstract

**Background:**

Neuroblastoma is one of the common extracranial tumors in children (infants to 2 years), accounting for 8 ~ 10% of all malignant tumors. Few special drugs have been used for clinical treatment currently.

**Results:**

In this work, herbal extract ginsenosides were used to synthesize fluorescent ginsenosides carbon nanodots via a one-step hydrothermal method. At a low cocultured concentration (50 µg·mL^− 1^) of ginsenosides carbon nanodots, the inhibition rate and apoptosis rate of SH-SY5Y cells reached ~ 45.00% and ~ 59.66%. The in vivo experiments showed tumor volume and weight of mice in ginsenosides carbon nanodots group were ~ 49.81% and ~ 34.14% to mice in model group. Since ginsenosides were used as sole reactant, ginsenosides carbon nanodots showed low toxicity and good animal response.

**Conclusion:**

Low-cost ginsenosides carbon nanodots as a new type of nanomedicine with good curative effect and little toxicity show application prospects for clinical treatment of neuroblastoma. It is proposed a new design for nanomedicine based on bioactive carbon nanodots, which used natural bioactive molecules as sole source.

**Supplementary Information:**

The online version contains supplementary material available at 10.1186/s12951-023-02023-w.

## Introduction

Traditional drugs usually treat diseases in molecular state. In fact, it is difficult for the drug molecules to penetrate the cell membranes and enter the cells in large quantities, which resulting in low efficacy [1–5]. With the rising researches of nanomedicine, it has found that nanomedicines with suitable sizes and properties can enter cells through endocytosis in large quantities, and efficiently exert their medicinal effects [1–3, 6]. Thus, researchers have designed various medical nanomaterials with different hybrid structures in the field of nanomedicine, such as nanosuspensions, nanoliposomes, metal nanoparticles, inorganic nanoparticles, and polymer nanoparticles [3–5, 7–10]. Yet, most of these reported medical nanomaterials with good therapeutic effects always require cumbersome production processes. Carriers with little drug activity in these nanomedicines often inevitably interact with biological matrix of organisms, resulting in biological toxicity [10, 11].

Over the past 10 years, the researches and applications of carbon nanodots (CDs) have developed rapidly in the field of nanomedicine [12–20]. CDs have certain advantages, including unique fluorescence properties, good water solubility, high cell uptake rates, and eco-friendly. More importantly, CDs that use carbon as the main substrate have extremely low biological toxicity, a simple preparation process, and can be made by a wide range of raw materials, for instance citric acid, glucose, amino acids, lemon juice and so on [12–15]. CDs are considered very suitable candidates for nanomedicine. Up to now, large amounts of CDs for nanomedicine are mainly focused on fluorescent markers, drug carriers, thermal sensitizer, photosensitizer and sonosensitizer [16–20]. When are used as carriers, CDs usually have little medicinal activities. The number of loaded drugs on CDs as well as their pharmacological effects are limited. When CDs are used as thermal sensitizer, photosensitizer or sonosensitizer, the structure and composition of the CDs system are usually complex. And the preparation procedures are correspondingly cumbersome, with high technical requirements and cost [19, 20].

Interestingly, some recent studies directly used bioactive molecules as the main source to prepare bioactive CDs [21–26]. These CDs displayed good disease treatment effects, and avoided the toxic interference caused by other chemical substances. For instance, Jiangong Liang et al. prepared glycyrrhizic acid carbon dots (Gly-CDs) with low toxicity by hydrothermal method with glycyrrhizic acid as the sole carbon source [21]. Compared with glycyrrhizic acid molecules and common CDs, Gly-CDs possessed much higher biological activity and inhibited a variety of viruses. However, this type of research system has not been paid more attention.

Botanicals have received numerous attention due to increasing interest in green and natural product worldwide. Many ingredients extracted from natural plants show low toxicity, good biological activity and medicinal effects [13, 27, 28]. For example, ginsenosides (GS), with unique tetracyclic triterpene structure, is an important active ingredient in ginseng- a precious medicinal plant. Various studies showed that GS can inhibit tumors, improve immunity, and regulate the nervous system [29–31]. Unfortunately, herbal extracts in molecular state also cannot be transported across cell membranes, leading to low cellular uptake and medicinal effects.

**Scheme 1:**
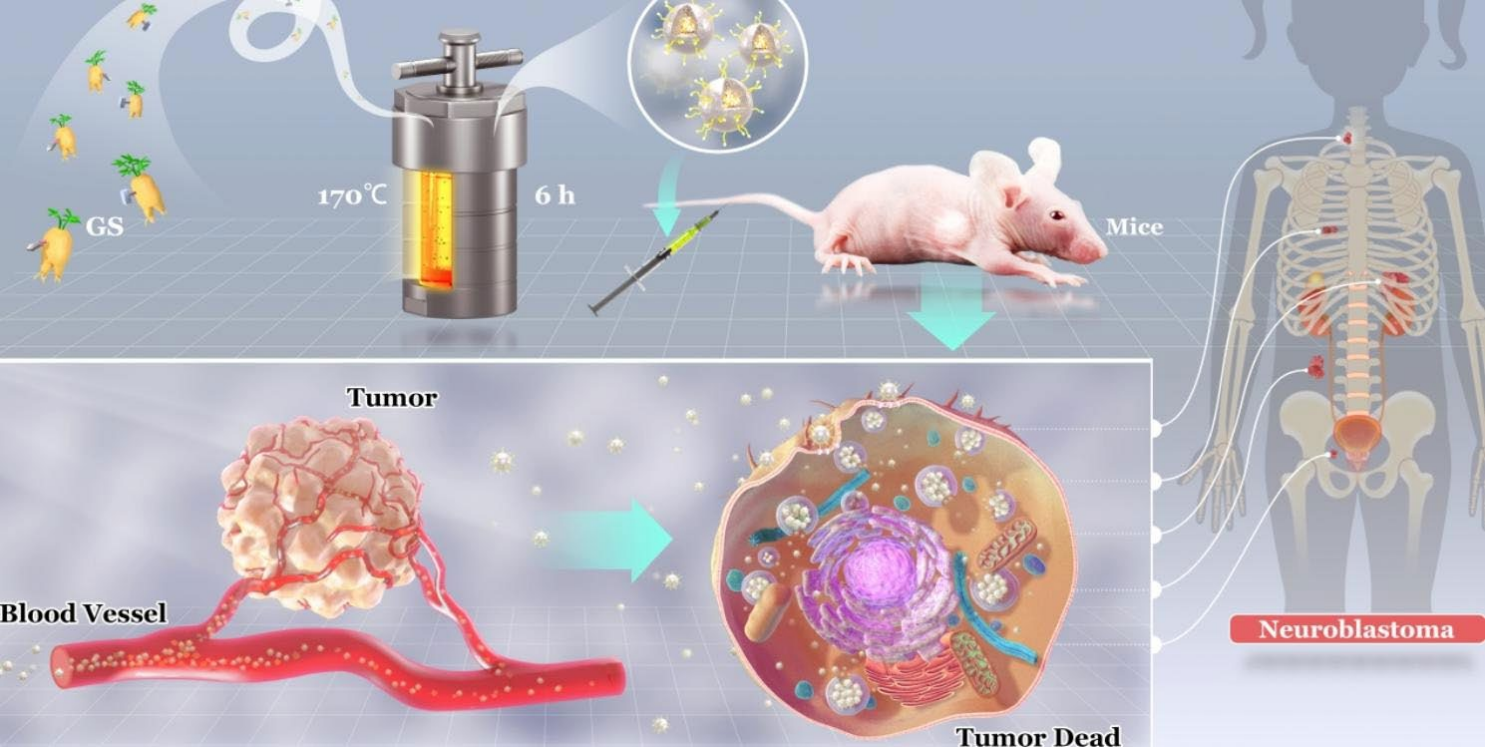
The synthetic route of GS-CDs and the schematic diagram of their inhibitory effect on human neuroblastoma

For the aim to elevate medical activity and avoid biological toxicity caused by other chemical substances during synthesis, ginsenosides carbon nanodots (GS-CDs) were prepared using a one-step hydrothermal method with GS as the only carbon source. The size of prepared GS-CDs was in the range of 2–4 nm, and the optimal excitation and emission wavelengths were ~ 360 and ~ 440 nm, respectively. Cell activity screening and apoptosis revealed that GS-CDs had good inhibitory effect on human neuroblastoma cells (SH-SY5Y). The in vivo animal experimental results further confirmed that GS-CDs had high inhibitory effects on neuroblastoma (Scheme 1).

Neuroblastoma is one of the common extracranial tumors in children. The average age of clinical diagnosis is infants to 2 years. Few special drugs have been used for clinical treatment currently, except several common chemotherapy drugs such as cisplatin and carboplatin [32, 33]. Obviously, certain groups of infants and young children often have severe side effects to these drugs. The clinical experience is very painful for these particular patients. In this study, we proved that the GS-CDs were non-toxic to the animal organs and showed few adverse reactions in the mice. Due to their low cost, simple preparation process, and good curative effect, the GS-CDs could have application prospects for the clinical treatment of human neuroblastoma in children.

## Experimental section

### Devices

The transmission electron microscopy (TEM) and high-resolution transmission electron microscopy (HRTEM) images of the GS-CDs were obtained using a Tecnai F20 electron microscope (FEI Company, Netherlands). Dynamic light scattering (DLS) measurement was carried out by a Zetasizer Nano-ZS (Malvern Instruments). We also used a Shimadzu UV-2550 ultraviolet-visible spectrophotometer to obtain the ultraviolet-visible absorption spectrum, and a Hitachi F-2700 fluorescence spectrophotometer to obtain the fluorescence spectra. The infrared spectra were obtained using a Vector 33 Fourier transform infrared spectrometer (Bruker, Germany). An FLS 920 steady-state/transient fluorescence spectrometer (Edinburgh, Scotland) was used to obtain the fluorescence lifetime and quantum yield. X-ray photoelectron spectroscopy was conducted on an ESCALAB 250 X photoelectron spectrometer (Thermo Scientific, USA). Mass spectra were analyzed by Q-Exactive high-resolution LC/MS analyzer (Thermo Scientific, USA).

### Materials

The GS (stem and leaf extraction, UV > 80%), D(+)-Glucose (HELP > 98%) and paraformaldehyde (4%) were obtained from Shanghai Yuanye Biotechnology Co., Ltd. (China). Cell counting kit-8 was obtained from Wuhan Boster Biological Technology Co., Ltd. (China). The FITC annexin V apoptosis detection kit I was from BD Biosciences Pharmingen (USA). DNA content quantitation assay (cell cycle) detection kit, anti-fluorescence attenuation sealing agent, 4’,6-diamidino-2-phenylindole dihydrochloride (DAPI), and BCA protein assay kit were obtained from Beijing Solarbio Science & Technology Co., Ltd. (China). The ProteinExt^®^ mammalian total protein extraction kit was obtained from Beijing TransGen Biotech Co., Ltd. (China). Ultra-sensitive enhanced chemiluminescence (ECL) kit was obtained from Shanghai Beyotime Biotech Co., Ltd. (China). Antibodies against Bax (no. ab32503), Bcl-2 (no. ab32124), Caspase-3 (no. ab32351), Caspase-10 (no. ab32155), β-actin (no. ab115777), and secondary antibodie (goat anti-rabbit, no. ab205718) were obtained from Abcam (England). The cisplatin injections (2 mL, 10 mg) were obtained from Yunnan Phytopharmaceutical Co., Ltd. (China). Dulbecco’s modified eagle medium (DMEM), Roswell park memorial institute medium (RPMI 1640), Dulbecco’s modified eagle medium/nutrient mixture F-12 (DMEM/F-12), penicillin-streptomycin solution, and certified fetal bovine serum (FBS) were obtained from VivaCell, Shanghai (China). Eagle’s minimum essential medium with Earle’s balanced salts (MEM-EBSS) was obtained from Hyclone (USA). The water used in all experiments was ultrapure water.

#### Preparation of GS-CDs

GS-CDs were synthesized according to a typical hydrothermal method [12–15]. First, 10 mL of GS aqueous solution (1.5 mg·mL^− 1^) was transferred into a 15 mL polytetrafluoroethylene lined autoclave and heated in an oven at 170 °C for 1–10 h with 1 h intervals. After reaction was complete, the obtained solution was coarsely filtered using a 0.22 μm polyethersulfone membrane to remove the larger products. Then, the filtered solution was added to an activated ultrafiltration tube (3000 MWCO), where it underwent centrifugal purification twice (5000 rpm, 15 min). Finally, the light-yellow GS-CDs solution was obtained at different reaction times (GS-CDs@1 h, GS-CDs@2 h, GS-CDs@3 h, GS-CDs@4 h, GS-CDs@5 h, GS-CDs@6 h, GS-CDs@7 h, GS-CDs@8 h, GS-CDs@9 h, and GS-CDs@10 h), and stored in a refrigerator at 4 °C.

### Preparation of the Glu-CDs

The glucose carbon nanodots (Glu-CDs) were prepared with a glucose aqueous solution (5 mg·mL^− 1^) via a typical hydrothermal reaction at 170 °C for 6 h, according to previous reports with minor modifications [14, 34]. The purification and storage processes of Glu-CDs were the same as GS-CDs.

### Preparation of GS@Glu-CDs

5 mL of 3 mg·mL^− 1^ GS aqueous solution was introduced into 10 mL of the Glu-CDs aqueous solution. Under nitrogen protection, the mixture was stirred in a water bath at 40 °C for 5 h. The purification and storage processes of GS@Glu-CDs were the same as the GS-CDs.

### Cell cultures

All cells were from an American type culture collection (ATCC, Manassas, VA, US). The human neuroblastoma (SH-SY5Y) and human renal epithelial (293T) cells were cultured in DMEM/F-12 medium, and human cervical cancer (HeLa) and mouse microglial (BV2) cells were cultured in DMEM medium. Human liver cancer (HepG2) cells were cultured in MEM-EBSS medium, and rat adrenal pheochromocytoma (PC12) and human normal liver (LO2) cells were cultured in RPMI-1640 medium. These media were supplemented with 10% FBS and 1% penicillin-streptomycin. All cells were cultured in a humidified incubator containing 5% CO_2_ at 37 °C.

### Testing in vitro cytotoxicity

CCK-8 method was used to detect toxicity effects of GS-CDs (GS-CDs@1 h, GS-CDs@2 h, GS-CDs@3 h, GS-CDs@4 h, GS-CDs@5 h, GS-CDs@6 h, GS-CDs@7 h, GS-CDs@8 h, GS-CDs@9 h, and GS-CDs@10 h) on various cells. Using GS-CDs vs. SH-SY5Y cells as an example, SH-SY5Y cells were cultured to the logarithmic growth phase, according to the appropriate cell density of 3000 ~ 5000 cells well^− 1^. Then, SH-SY5Y cells were transferred and cultured into a 96-well cell culture plate. Afterward, 180 µL of cell suspension per well was placed in an incubator containing 5% CO_2_ at 37 °C. When cells were completely attached to the cell bottle wall, we added 20 µL of GS-CDs at different concentrations. Next, GS-CDs concentrations for the cells in each well were 0, 10, 20, 30, and 50 µg·mL^− 1^ (each concentration had 6 multiple wells). Then, culturing was continued. After GS-CDs and SH-SY5Y cells were incubated for 48 h, 20 µL of CCK-8 was added to each well. The 96-well cell culture plate was placed in incubator for 30 min, and optical density (OD) was measured at 450 nm with a microplate reader (infinite M200 PRO from TECAN, Switzerland). SH-SY5Y cell viability was calculated according to the OD value.

The CCK-8 method was also used to detect viability of the other four cells (HeLa, BV2, PC12, and HepG2) after incubation with GS-CDs; the viability of two cells (293T and LO2) after incubation with GS-CDs@6 h; the viability of SH-SY5Y cells after incubation with GS, Glu-CDs, and GS@Glu-CDs; the viability of SH-SY5Y cells after incubation with GS-CDs@6 h for 6, 12, 24, and 48 h. All the experimental procedures were the same as above.

### Cellular fluorescence imaging

A circle microscope cover glass was placed on a 6-well cell culture plate. Then, 2 mL of SH-SY5Y cell suspension with about 1 × 10^5^ cells well^− 1^ was added to the 6-well cell culture plate. The plate was incubated for 24 h in an incubator containing 5% CO_2_ at 37 °C. The medium in the wells was replaced with a medium containing GS-CDs@6 h solution, and the GS-CDs concentration in each well was controlled to 100 µg·mL^− 1^. Then, GS-CDs@6 h with the SH-SY5Y cells were incubated for 30 min at 2, 4, 6, 12, 24, and 48 h. Afterward, the cell morphology was fixed with 4% paraformaldehyde solution, and the cell nucleus was stained with DAPI. For the 6-well cell culture plate, glass slide was removed, and buckled by a glass slide mounted with 10 µL of anti-fluorescence attenuation solution. Cell fluorescence images were captured by a fluorescence microscope (EVOS FL auto automatic fluorescence inverted microscope system, Life Technologies, USA).

### Determination of intracellular distribution of GS-CDs by TEM

Cells (SH-SY5Y, 293T, LO2) in logarithmic growth phase were incubated with GS-CDs at same concentration for 0, 6, 12 and 24 h. After digestion and centrifugation, the cell pellets were fixed, dehydrated, embedded, then sectioned and stained. At last, the TEM images of the cells were obtained [35].

### Analysis of apoptosis and cell cycle by flow cytometry

SH-SY5Y cells were cultured to the logarithmic growth phase in a 6-well cell culture plate at a cell density of 1 × 10^5^ cells well^− 1^. Then, they were incubated for 24 h in an incubator containing 5% CO_2_ at 37 °C. The medium in the culture plates was replaced with medium containing GS-CDs@6 h, and its final concentration in each well was 0, 10, 20, 30, and 50 µg·mL^− 1^.

After incubated for 48 h, the cells were harvested and washed twice with PBS. FITC coupled with an annexin-v apoptosis detection kit I was used to detect apoptosis of the SH-SY5Y cells. The stained cells were analyzed by flow cytometry (BD FACSCalibur, BD Biosciences Pharmingen, USA), where a total of 1 × 10^4^ cells were counted for each sample.

After incubated for 48 h, the cells were harvested in a centrifuge tube and fixed with 500 µL of 70% ice-cold ethanol at 4℃ for 4 h. The cells were washed twice with PBS and then DNA content quantitation assay detection kit was used to detect cell cycle of SH-SY5Y cells. The stained cells were analyzed by flow cytometry, where a total of 1 × 10^4^ cells were counted for each sample.

### Western-blot

Protein expression was detected by western-blot. First, the steps of cell treatment were the same as the above cell cycle and apoptosis procedures. Then cells were harvested and washed twice with PBS. The cells were then suspended in protein extraction buffer containing protease inhibitors, and lysed on ice for 30 min. The supernatant was collected after centrifugation at 12,000 × g for 10 min, and the protein content was measured using the BCA protein assay kit. 10–15% sodium dodecyl sulphate-polyacrylamide gel (SDS-PAGE) was performed for equal amount of protein per sample followed by transfer to a PVDF membrane (Roche, UK), which were then soaked in blocking buffer (5% skimmed milk) for 1 h. The membranes were then incubated with relevant primary antibodies overnight at 4℃, followed by incubation with the appropriate horseradish peroxidase-conjugated secondary antibodies for 1 h at room temperature. Finally, the signals were visualized using enhanced chemiluminescence. β‑actin was used as reference protein.

### In vivo experiments to investigate neuroblastoma inhibition

Four-week-old male BALB/c nude mice were purchased from Beijing Huafukang Biological Technology Co., Ltd. and raised in a specific-pathogen-free laboratory. To evaluate the abilities of the GS-CDs to inhibit human neuroblastoma cells in vivo, SH-SY5Y cells were inoculated into the right axilla of male BALB/c nude mice (5-week-old), at a loading of ~ 3 × 10^6^ cells per mouse, to establish a subcutaneous neuroblastoma mouse model. After average volume of axillary neuroblastoma in the nude mice met or exceeded ~ 60 mm^3^, the tumor-bearing mice were randomly divided into 3 groups (n = 3): the model group (injection of corresponding doses of physiological saline into the tail vein, according to the mouse weight, once every 36 h), cisplatin group (intraperitoneal injection of corresponding doses of 5 mg·kg^− 1^ of cisplatin according to the mouse weight, once every 4 days), GS-CDs group (injection of corresponding doses of 8 mg·kg^− 1^ of the GS-CDs into the tail vein, according to the mouse weight, once every 36 h) [36, 37].The day that administration started was set as the first day of the treatment cycle. Starting from the first day, we recorded the weight of the mice and took photos of the tumors once every two days. We used a small animal Micro-CT imaging system (Quantum GX of PerkinElmer, USA) to scan the mice in the model, cisplatin, and GS-CDs groups, once every four days. We then monitored tumor growth and accurately measured the tumor volume. After the 13th day of treatment, all of the nude mice were euthanized for anatomy and histopathological analyses.

To further investigate the in vivo biosafety of GS-CDs, we randomly divided healthy male BALB/c nude mice of 5-week-old into another two groups (n = 3): Control group (injected with saline via tail vein) and Control + GS-CDs group (tail vein injection of 8 mg·kg^− 1^ of GS-CDs). The dosing cycle and times of dosing were the same as above, and the weights of the mice were recorded. After the 13th day of treatment, all nude mice were sacrificed for dissection and histopathological analysis.

## Results and discussion

### Characterization of GS-CDs

**Fig. 1:**
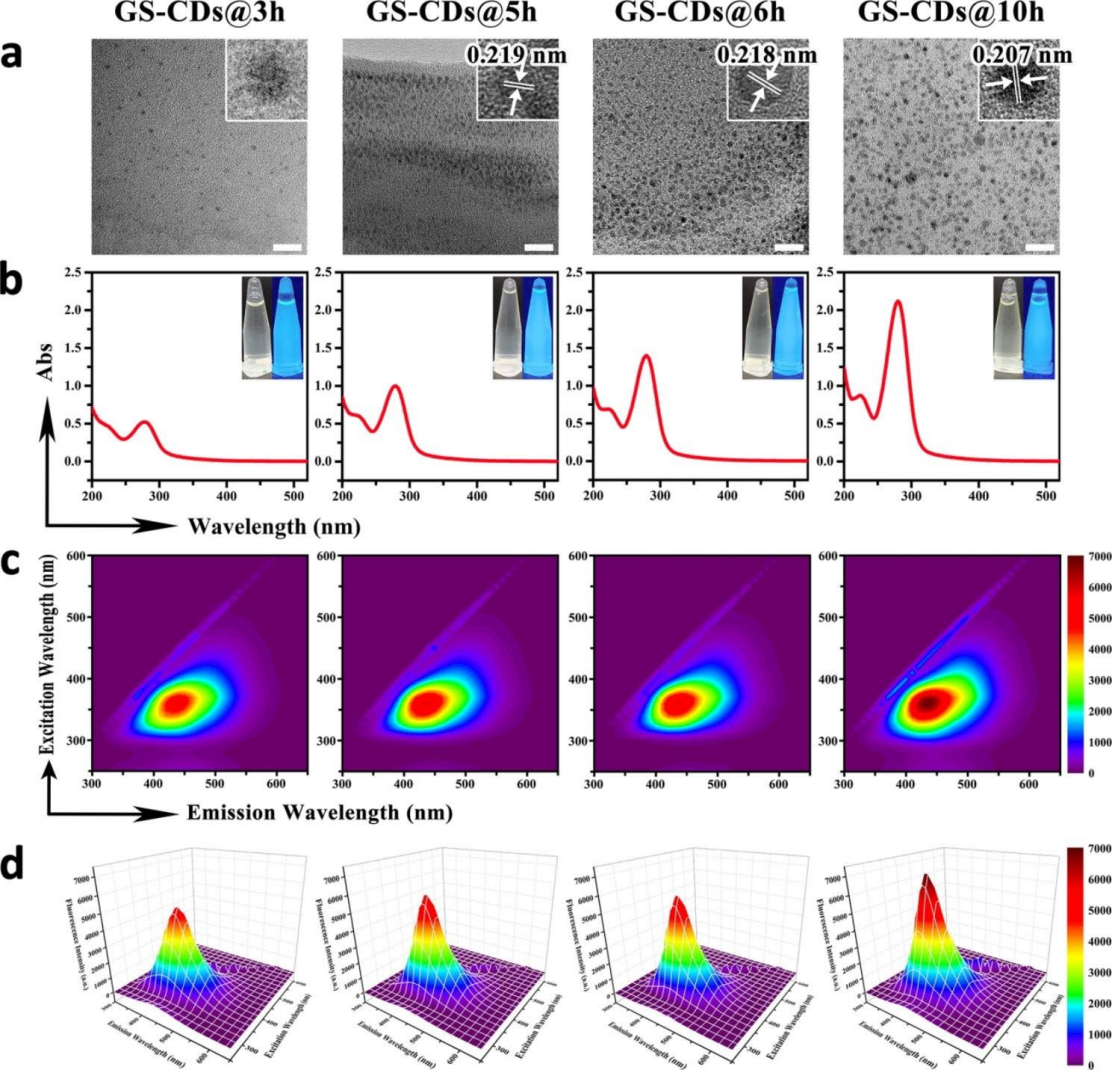
Characterization of GS-CDs under different synthesis conditions. **a** TEM images of the prepared GS-CDs@3 h, GS-CDs@5 h, GS-CDs@6 h, and GS-CDs@10 h samples, respectively, where the HRTEM images inserted in the upper right corner show the crystal lattices of the four GS-CDs. The scale bars were 20 nm. **b** UV absorption spectra of the prepared GS-CDs@3 h, GS-CDs@5 h, GS-CDs@6 h, and GS-CDs@10 h samples, where the insets show the photos of the four GS-CDs under sunlight (left) and UV light at 365 nm (right). **c** Contour maps showing the three-dimensional fluorescence spectra of GS-CDs@3 h, GS-CDs@5 h, GS-CDs@6 h, and GS-CDs@10 h, respectively. **d** Isometric projections of the three-dimensional fluorescence spectra of GS-CDs@3 h, GS-CDs@5 h, GS-CDs@6 h, and GS-CDs@10 h.

A series of GS-CDs at 170 °C using GS as the single reactant at different reaction times was synthesized, from 1 to 10 h, via normal hydrothermal synthesis. High-resolution transmission electron microscopy (HRTEM) was used to characterize the size and surface morphologies of GS-CDs@3 h, GS-CDs@5 h, GS-CDs@6 h, and GS-CDs@10 h at different hydrothermal reaction times of 3, 5, 6, and 10 h, respectively. All of the GS-CDs showed good dispersity with even sizes, where the diameter of GS-CDs@3 h was concentrated at 2.95 ± 0.72 nm, the diameter of GS-CDs@5 h was concentrated at 2.75 ± 0.66 nm, the diameter of GS-CDs@6 h was concentrated at 3.00 ± 0.64 nm, and the diameter of GS-CDs@10 h was concentrated at 3.24 ± 0.62 nm (Fig. 1a). The HRTEM images shown in the upper right corner of Fig. 1a presented the crystal lattices of the four prepared GS-CDs. GS-CDs@3 h had a mere crystal lattice, possibly due to the short reaction time and incomplete nanostructure. GS-CDs@5 h had a certain lattice structure with a lattice spacing of 0.219 nm. GS-CDs@6 h and GS-CDs@10 h showed obvious lattices, with lattice spacings of 0.218 and 0.207 nm, respectively. This implied that with prolonged reaction time, the self-assembly of the GS molecules became more regular, and the structures of the GS-CDs became more complete. In UV absorption spectra, the GS aqueous solution had weak UV absorption, while the GS-CDs showed strong absorption at a wavelength of 280 nm (Fig. 1b and Additional file 1: Figure S1-1). The longer the reaction time, the higher the UV absorption peak. Combined with IR spectroscopy analysis, the absorption peak at 280 nm was mainly derived from the vibration and rotation of C = O (Additional file 1: Figure S1-2). The inset (Fig. 1b) is the photos of the four prepared GS-CDs under sunlight (left) and UV light at 365 nm (right), respectively. The GS-CDs with different reaction times had strong blue-green fluorescence, and the brightness increased slightly with increased reaction time.

In the contour map of the three-dimensional fluorescence spectra of the four prepared GS-CDs with different reaction times, there was only one substance with fluorescent emission in each system, indicating a single product (Fig. 1c). As shown in the isometric projection of the three-dimensional fluorescence spectra (Fig. 1d), all the four GS-CDs had excitation dependence. Under the optimal excitation wavelength of ~ 360 nm, the optimal emission wavelength was ~ 440 nm for the four GS-CDs. The longer the reaction time, the higher the obtained fluorescence intensity of the GS-CDs. In the fluorescence attenuation curves of the four GS-CDs (Additional file 1: Figure S1-3), three fluorescence lifetimes (τ) were obtained, after fitted according to a third-order decay exponential function. It indicated that the CDs had three fluorescence centers. The quantum yields of GS-CDs@3 h, GS-CDs@5 h, GS-CDs@6 h, and GS-CDs@10 h were ~ 0.19%, ~ 0.14%, ~ 0.19%, and ~ 0.22%, respectively. X-ray photoelectron spectroscopy (XPS) was used to further characterize and analyze the elemental compositions and functional groups of the four GS-CDs. In the XPS spectra, all the four GS-CDs had two main peaks at 285.1 and 531.6 eV, corresponding to the C 1s and O 1s elements, respectively (Additional file 1: Figure S1-4). The C 1s band had three peaks near 288.5, 285.6, and 284.5 eV, which was attributed to the carbon peaks related to C = O, C-O, and C-C. The O 1s band had two peaks near 534.3 and 532.6 eV, which were attributed to the oxygen peaks of C = O and C-O.

Studies have shown that the content in typical conjugated fluorophore groups, such as carbon-carbon double bonds, carbon-carbon triple bonds, or carbon-based π electrons in GS molecules is very low [29]. The main sources of UV absorption and fluorescence emission of the prepared GS-CDs system did not consist of these classic conjugated structures. The appearance and increase in UV and fluorescence could be attributed to the cross-linking enhanced emission (CEE) effect [38]. Under a hydrothermal condition of 170 °C, and with rapid collisions between the particles, the free GS were cross-linked through a buckling reaction, forming a carbon nucleus. During the reaction, the main tetracyclic triterpene structure of GS molecule showed few changes, according to the mass spectra analysis of GS, GS-CDs@3 h, GS-CDs@5 h, GS-CDs@6 h, and GS-CDs@10 h (Additional file 1: Figure S1-5 and Table S1-1). The active site beside the main structure reacted with H_2_O, and the number of C = O groups could increase in the system (Additional file 1: Figure S1-2). With prolonged reaction time (3, 5, 6, and 10 h), the buckle reaction, as well as the degree of carbonization increased. The structures of the CDs became denser and more orderly (Fig. 1a), and more C = O and C-O groups in the system became fixed. Their vibrations and rotations were limited, resulting in increased radiation transition. Thus, the UV absorption and fluorescence emissions of the GS-CDs generated by non-classical conjugation were enhanced [38, 39].

### Inhibitory effect of GS-CDs on cells

The above results demonstrated that under hydrothermal conditions, and within a certain reaction time, the GS molecules could self-assemble to form CDs. When reaction temperature (170 °C) was far below the threshold temperature for the ring-opening reaction, the main tetracyclic triterpene structure of GS could not be destroyed during the self-assembly process. Thus, we proposed the self-assembled GS-CDs could retain biomedical activity of GS to a certain extent. On this basis, cell counting kit-8 (CCK-8) method was used to estimate cell viabilities of human neuroblastoma cells (SH-SY5Y), human cervical cancer (HeLa), mouse microglial (BV2), rat adrenal pheochromocytoma (PC12), and human hepatoma (HepG2) cells. They were incubated with different GS-CDs (GS-CDs@1 h, GS-CDs@2 h, GS-CDs@3 h, GS-CDs@4 h, GS-CDs@5 h, GS-CDs@6 h, GS-CDs@7 h, GS-CDs@8 h, GS-CDs@9 h, GS-CDs@10 h) for 48 h. The cell viabilities of HeLa, BV2, PC12, and HepG2 cells did not change significantly after incubation with GS-CDs under the same conditions (Additional file 1: Figure S2-1, S2-2, S2-3, and S2-4).

**Fig. 2:**
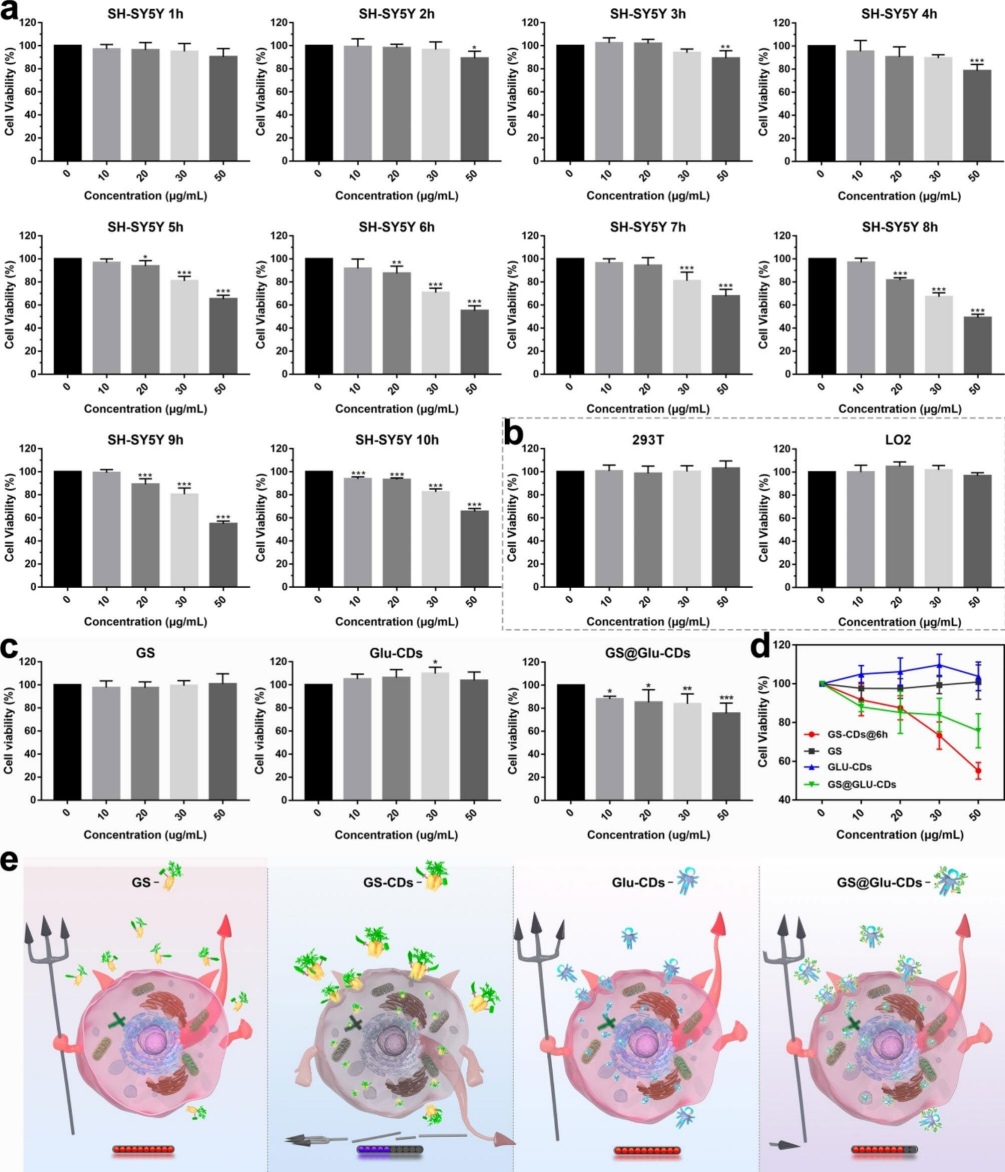
Inhibitory effect of GS-CDs prepared from 1–10 h and different CDs on SH-SY5Y cells. **a** In vitro cytotoxicity profiles of GS-CDs@1 h, GS-CDs@2 h, GS-CDs@3 h, GS-CDs@4 h, GS-CDs@5 h, GS-CDs@6 h, GS-CDs@7 h, GS-CDs@8 h, GS-CDs@9 h, and GS-CDs@10 h on SH-SY5Y cells. **b** In vitro cytotoxicity profiles of GS-CDs@6 h on 293T and LO2 cells. **c** In vitro cytotoxicity profiles of GS, Glu-CDs, and GS@Glu-CDs on SH-SY5Y cells. **d** Statistical chart summarizing the in vitro cytotoxicity profiles of GS-CDs@6 h, GS, Glu-CDs, and GS@Glu-CDs on SH-SY5Y cells. **e** Schematic images showing the cytotoxicity profiles of GS, GS-CDs, Glu-CDs, and GS@Glu-CDs on SH-SY5Y cells. Data were mean ± s.d. (n = 6). *p < 0.05, **p < 0.01 and ***p < 0.001 relative to 0 µg·mL^− 1^, as analyzed by one-way analysis of variance (ANOVA).

Note that GS-CDs had few inhibitory effects on human neuroblastoma cells (SH-SY5Y), when reaction time was 1–4 h (Fig. 2a). At a GS-CDs dosage of 50 µg·mL^− 1^, some cytostatic effects appeared. When reaction time increased to 5 h, GS-CDs started to display obvious inhibitory effect on SH-SY5Y cells. At a reaction time of 6 h, GS-CDs had a stronger inhibitory effect on SH-SY5Y cells. The cell viability rates were ~ 87.56%, ~ 73.26% and ~ 55.05% at GS-CDs dosing concentrations of 20, 30 and 50 µg·mL^− 1^, respectively. The higher the dose, the lower the cell viability. This suggested that the toxic effects of GS-CDs on SH-SY5Y cells were concentration-dependent. When reaction time extended to 7–10 h, the inhibitory effects of GS-CDs on SH-SY5Y cells were similar to GS-CDs@6 h. The two other repeated experiments were mostly identical (Additional file 1: Figure S2-5). The experimental results showed the prepared GS-CDs had a very effective inhibitory effect on SH-SY5Y cells. A low concentration of GS-CDs (50 µg·mL^− 1^), their inhibition rate of SH-SY5Y cells achieved ~ 45.00%. Since reaction temperature (170 °C) was far below the threshold temperature of the ring-opening reaction, the main tetracyclic triterpenoid structure of GS could not be destroyed. Therefore, we proposed that GS-CDs retained the pharmaceutical activity of the carbon-sourced GS, and introduced largest number of active substances into SH-SY5Y cells through endocytosis, with best efficacy. In addition, since the substance of GS-CDs was natural plant essence, they showed good non-cytotoxicity to ordinary cells, such as human renal epithelial (293T) cells and human normal liver (LO2) cells (Fig. 2b).

Considering the obviously higher inhibitory of GS-CDs on SH-SY5Y than other cells (HeLa, BV2, PC12, and HepG2 cells), there were two speculations: (i) There are differences of active receptor in membrane surface among different tumor cells. GS-CDs may bind to certain specific receptors on the surface of SH-SY5Y cells, activating the tumor apoptosis pathway and thus exerting inhibitory effects. (ii) Gene level effects: The occurrence of tumors is the result of multiple genes, multiple steps, and multiple mutations. Mutations in different genes with different intensities lead to the formation of different tumors. The addition of GS-CDs may change the structure of genetic DNA of SH-SY5Y cells, reverse the genetic characteristics of SH-SY5Y cells, and inhibit the tumor effectively.

### Different inhibitory effect on SH-SY5Y cells

Furthermore, under the same conditions we compared the inhibition efficiency of GS molecules and CDs with different structure/composition on SH-SY5Y cells, via in vitro cytotoxicity experiments (Fig. 2c and d, and 2e). During the same concentration range, GS had no inhibitory effects on SH-SY5Y cells. It was inferred that GS in molecular state could not be effectively taken up via cell transport and provided effective drug effect. On contrary, due to the nano-size and nanostructure, GS-CDs could be taken up by cells in large numbers through endocytosis. Thus, GS-CDs showed a much higher inhibitory efficiency on SH-SY5Y cells than GS molecules. Second, molecule glucose of little medicinal activity was used to prepare common glucose carbon nanodots (Glu-CDs) via a similar hydrothermal reaction [14, 34, 40, 41]. When concentration of Glu-CDs was 50 µg·mL^− 1^, viability of SH-SY5Y cells was weakly increased. It indicated common Glu-CDs synthesized by non-drug molecules had little drug activity. Lastly, GS was loaded onto the carrier Glu-CDs through supramolecular forces. Then drug molecule GS composite carbon nanodots (GS@Glu-CDs) were obtained (Additional file 1: Fig. S2-6). At the same concentration of 50 µg·mL^− 1^, inhibitory effect (~ 75.69%) of GS@Glu-CDs on SH-SY5Y cells became obviously but much less than that of GS-CDs. It can be explained that after loaded on Glu-CDs, drug molecule GS could enter cell interior through endocytosis and had a damaging effect on SH-SY5Y cells. However, because of the nano-size of Glu-CDs, the loaded quantity of bioactive GS was limited, as well as their inhibitory effect on SH-SY5Y cells. Thus, the construction of nanodrug CDs composed of drug activity molecules could open up new research ideas and application directions for nanomedicine.

### Intracellular distribution of GS-CDs in SH-SY5Y cells with time

**Fig. 3:**
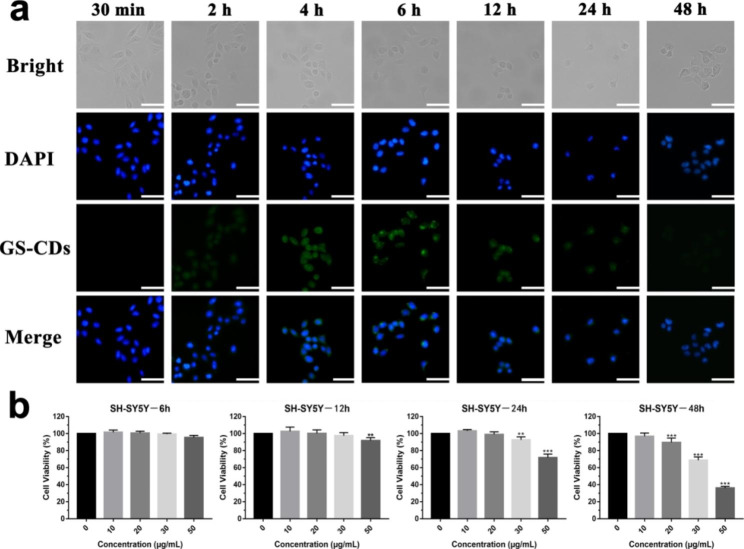
Fluorescent intracellular distribution, and cytotoxicity of GS-CDs in SH-SY5Y cells with time. **a** Fluorescence microscope images of the SH-SY5Y cells and GS-CDs (100 µg·mL^− 1^) incubated at different times (30 min, and 2, 4, 6, 12, 24, and 48 h). The scale bars were 50 μm. **b** In vitro cytotoxicity profiles of the SH-SY5Y cells after incubating with the GS-CDs at different times (6, 12, 24, and 48 h). Data were mean ± s.d. (n = 6). *p < 0.05, **p < 0.01 and ***p < 0.001 relative to 0 µg·mL^− 1^, as analyzed by one-way ANOVA.

Because GS-CDs prepared for 6–10 h showed similar inhibitory effects on SH-SY5Y cells, GS-CDs@6 h were chosen as the representative nano-drug, and were denoted as GS-CDs in the follow-up experiments. To further explore the inhibitory effect of GS-CDs on SH-SY5Y cells, entry of GS-CDs into cells at different incubation times was monitored by a fluorescent inverted microscope (Fig. 3a). Accompanied by its effective medicinal effects, the fluorescent GS-CDs system did not need to introduce other fluorescent substances for tracking and labeling. With prolonged incubation time, SH-SY5Y cells gradually shrank and became round. The SH-SY5Y cells gradually became apoptotic with increased time by the inhibition of GS-CDs. In the fluorescence field, GS-CDs entered cells and were mainly concentrated in the cytoplasm. Between 30 min and 6 h, fluorescence intensity of the system gradually increased, indicating the internalized number of GS-CDs increased with time. Between 6 and 12 h, fluorescence intensity of the system decreased. This was possibly due to the reactions and structure destruction of GS-CDs within the cells. Fluorescence intensity of the system was very weak after incubating for 24 h. By 48 h, little fluorescence was detected in the system. While GS-CDs played a medicinal role in the cells, they could also be metabolized out through exocytosis. Fluorescence intensity changes in the incubation system indicated that inhibitory effect of GS-CDs on SH-SY5Y cells was time-dependent.

### Cytotoxicity of GS-CDs in SH-SY5Y cells with time

The exact viability changes of SH-SY5Y cell after incubating with GS-CDs for different times were investigated (Fig. 3b). When incubated for 6 and 12 h, the GS-CDs had a minimal inhibitory effect on the SH-SY5Y cells. When incubated for 24 h, at higher GS-CDs concentrations (50 µg·mL^− 1^), the inhibitory effect appeared. After 48 h of incubation, GS-CDs showed a strong inhibitory effect on the SH-SY5Y cells, which was essentially consistent with the results above (Fig. 2a).

Bio-TEM analysis was conducted to compare the cellular uptake and intracellular distribution of GS-CDs in SH-SY5Y, 293T and LO2 cells, respectively (Additional file 1: Figure S3-1). Many GS-CDs entered all the cells and mainly existed in cytoplasm from 6 to 24 h. At 24 h, GS-CDs were still abundant in SH-SY5Y cells. In that case, some cells shrank, some cell membrane was broken, and some cell shape was destroyed. It proved that GS-CDs inhibited SH-SY5Y cells effectively. Within 6-12 h, GS-CDs also entered into 293T and LO2 cells, but little GS-CDs could be found at 24 h. It implied GS-CDs were basically metabolized within 24 h in 293T and LO2 cells. What was more, 293T and LO2 cells had normal morphology and good growth status at 24 h. It suggested that GS-CDs had little toxic effect on normal cells. Via the bio-TEM analysis, it can be inferred GS-CDs with hydrophilic surface functional groups were able to enter all these cells. GS-CDs possessed a high specific inhibitory effect on SH-SY5Y cells and little inhibitory effect on other cells.

### Apoptosis and cell cycle of SH-SY5Y cells induced by GS-CDs

**Fig. 4:**
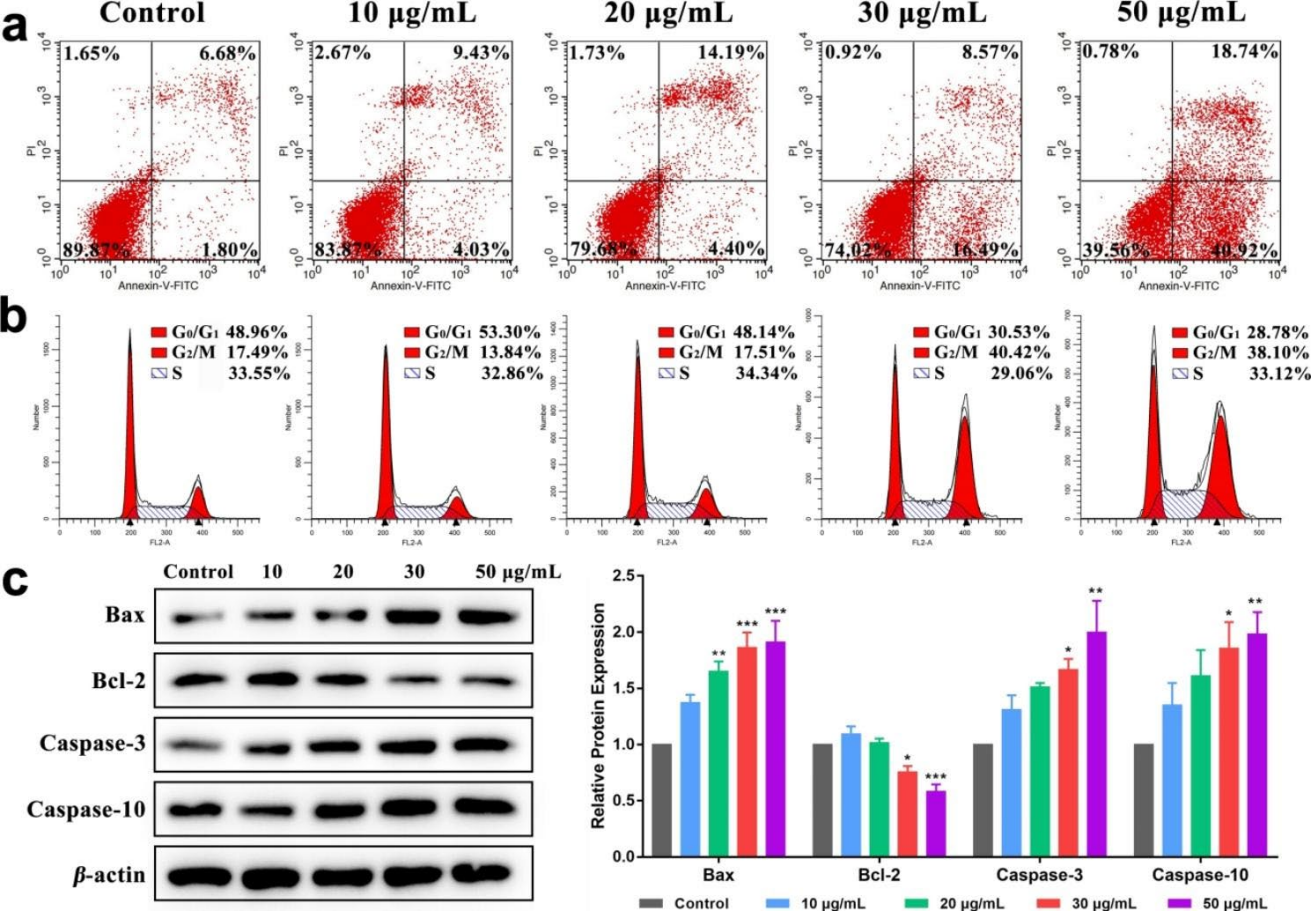
Apoptosis, cell cycle and expression of apoptosis-related proteins of SH-SY5Y cells induced by GS-CDs. **a** Apoptosis and **b** cell cycle charts of SH-SY5Y cells after incubating with different concentrations of GS-CDs (10, 20, 30, and 50 µg·mL^− 1^) for 48 h. **c** Effects of corresponding different concentrations of GS-CDs on the expression of apoptosis-related proteins in SH-SY5Y cells

Flow cytometry was used to detect apoptosis and cell cycle of SH-SY5Y cells after incubation at different GS-CDs concentrations after 48 h (Fig. 4a). When GS-CDs concentrations was 20, 30, and 50 µg·mL^− 1^, apoptosis rate of SH-SY5Y cells was ~ 18.59%, ~ 25.06%, and ~ 59.66%, respectively. The higher the administration concentration, the more significant the apoptosis rate. It demonstrated when GS-CDs were administered at high concentrations (for example, 50 µg·mL^− 1^), and at a sufficient incubation time (48 h), an inhibition effect on SH-SY5Y cells could be exerted.

When concentrations of GS-CDs were 10 and 20 µg·mL^− 1^, the cell cycle of SH-SY5Y cells changed a little (Fig. 4b). When concentration of GS-CDs was increased to 30 and 50 µg·mL^− 1^, SH-SY5Y cells in G0/G1 phase decreased to ~ 30.53% and ~ 28.78%, respectively. While in G2/M phase, SH-SY5Y cells increased to ~ 40.42% and ~ 38.10%, respectively. It indicated that GS-CDs could induce G2/M phase arrest of SH-SY5Y cell cycle.

### Effect of GS-CDs on the expression of apoptosis-related proteins in SH-SY5Y cells

The above apoptosis and cell cycle of SH-SY5Y cells showed that GS-CDs may inhibit tumor growth by inducing tumor cell apoptosis. Thus, we chose to detect the expression of apoptosis-related proteins in SH-SY5Y cells (Fig. 4c). Bax and Bcl-2 are homologous water-soluble related proteins, which promote apoptosis in cells. Bax can antagonize the protective effect of Bcl-2. Research showed that when the ratio of Bcl-2/Bax decreased, it can increase the permeability of mitochondrial membrane, activate caspase apoptosis pathway and induce cell apoptosis [42]. Among them, caspase-10 is the downstream signal molecule of mitochondria that mediates apoptosis, and caspase-3 is the executor of apoptosis. Cell death is inevitable after caspase-3 is activated [43]. Western-blot results showed that compared with the control group, when the drug concentration was 30 and 50 µg·mL^− 1^, the protein contents of Bax, caspase-3 and caspase-10 increased significantly (P < 0.05, P < 0.01, P < 0.001). The content of Bcl-2 protein decreased significantly (P < 0.05, P < 0.001). The above results suggested that apoptosis of SH-SY5Y cells induced by GS-CDs may be through inhibiting the up-regulation of Bcl-2, activating the caspase apoptosis pathway, activating and up-regulating the expression of apoptosis-related proteins caspase-3 and caspase-10. Finally, it caused SH-SY5Y cell death.

#### High inhibitory effect of GS-CDs on neuroblastoma and toxicity evaluation in vivo

**Fig. 5:**
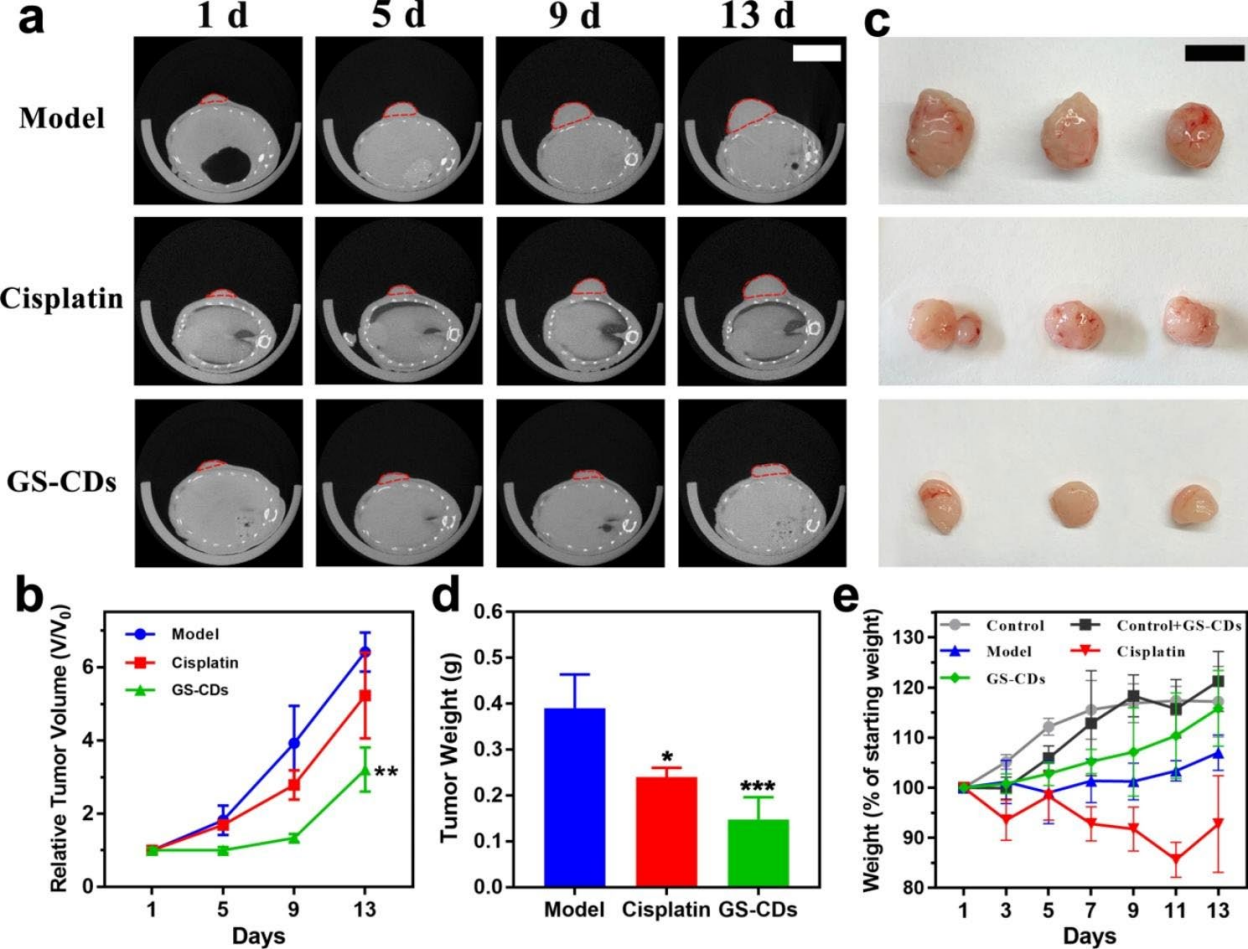
Inhibitory effect of GS-CDs on neuroblastoma in vivo. **a** Tumor growth of the mice tumors in the model (top), cisplatin (middle), and GS-CDs (bottom) group on the 1st, 5th, 9th, and 13th days as observed by CT scans (n = 3), where the scale bar was 1 cm. **b** Statistic charts showing the relative tumor volumes of the mice in the model, cisplatin and GS-CDs group, as calculated by the CT scans of the largest tumor sections. Data were mean ± s.d. (n = 3). **c** Photos of the dissected mice tumors in the model (top), cisplatin (middle) and GS-CDs (bottom) group at 13th day (n = 3), where the scale bar was 1 cm. **d** Statistic chart showing the tumor weight of the mice in the model, cisplatinand GS-CDs group. **e** Statistical weight diagrams of the mice in control, control + GS-CDs, model, cisplatin, and GS-CDs groups. Data were mean ± s.d. (n = 3). *p < 0.05, **p < 0.01 and ***p < 0.001 relative to the model group, as analyzed by one-way ANOVA.

To evaluate the inhibited ability of GS-CDs on human neuroblastoma in vivo, we employed BALB/c nude mice bearing human neuroblastoma tumors as the animal model. The antineoplastic cisplatin for the clinical treatment of neuroblastoma was administered to tumor-bearing mice as the positive group. The real-time photos of mice growth in the model, cisplatin, and GS-CDs group had been recorded carefully (Additional file 1: Figure S5-1, S5-2, and S5-3). Using a small animal CT imaging system, CT scans were performed to observe the growth of the mice tumors on the 1st, 5th, 9th, and 13th days during one experiment cycle (Fig. 5a). The statistic relative tumor volume of the model, cisplatin, and GS-CDs group, was calculated by the CT scans of the largest tumor sections (Fig. 5b). With time, the tumor growth trend of model group was obvious, and tumor growth in cisplatin group was slower than in the model group. The tumor growth rate of mice in GS-CDs group was the slowest and the tumor volume was the smallest. At the end of 13th day of experimental period, mice in each group were dissected and tumor comparisons were made (Fig. 5c). The tumor volume and statistic tumor weights of the GS-CDs group were significantly smaller than the other two groups (Fig. 5d). The above data showed that the prepared GS-CDs could effectively inhibit the growth of human neuroblastoma and produce substantial curative effects in vivo.

**Fig. 6:**
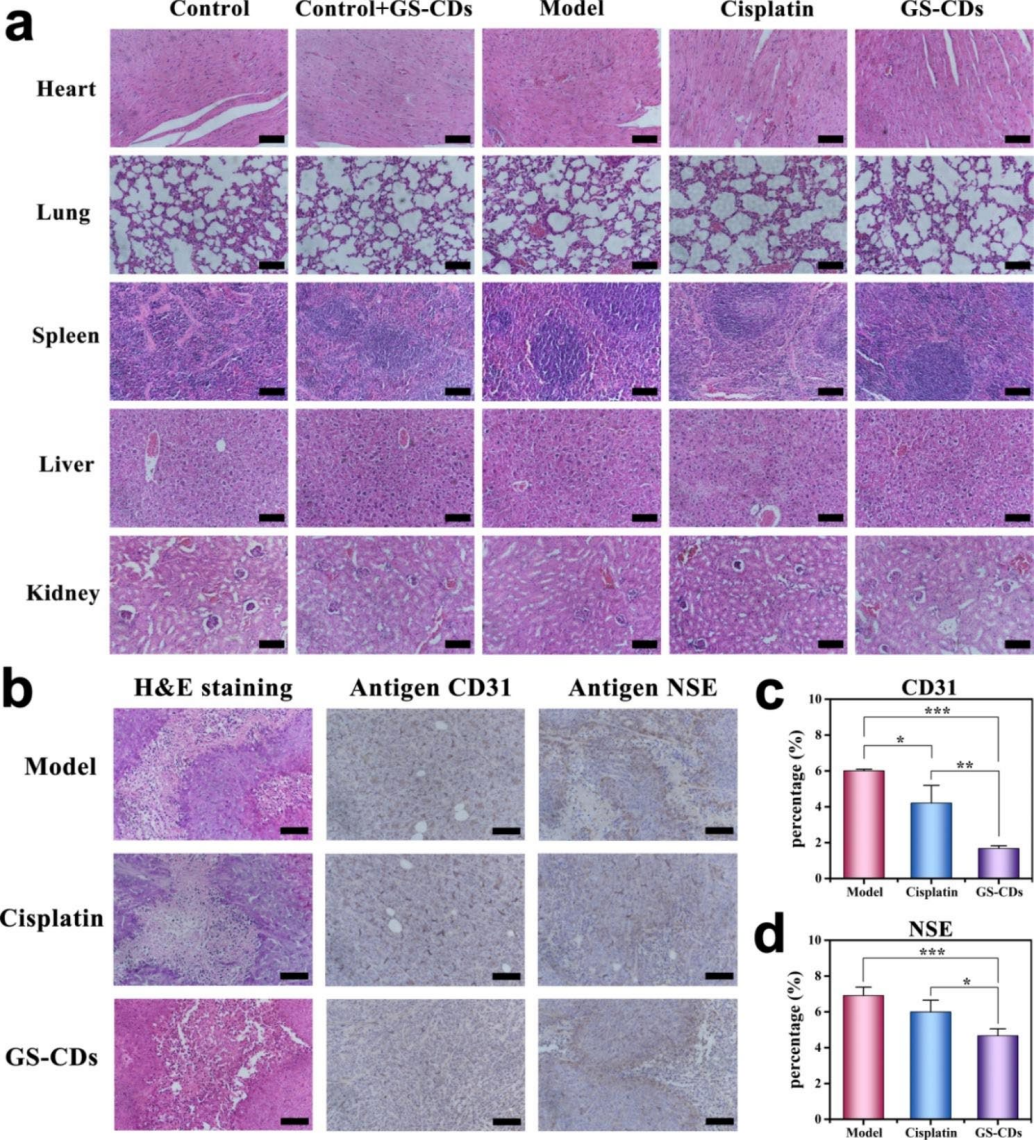
Stained sections of main organs and neuroblastoma tissues of mice. **a** H&E staining diagrams of the main organs (heart, lungs, spleen, liver, and kidneys) of mice in the control, control + GS-CDs, model, cisplatin, and GS-CDs groups, where the scale bars were 100 μm. **b** H&E (left), antigen CD31 (middle), and antigen NSE (right) stained neuroblastoma tissues of mice at 13th day in the model (top), cisplatin (middle) and GS-CDs (bottom) group, where the scale bars were 100 μm. **c** Statistic comparison chart of the antigen CD31 immunohistochemical stained neuroblastoma tissues of the mice in the model, cisplatin and GS-CDs group. **d** Statistic comparison chart of the antigen NSE immunohistochemical stained neuroblastoma tissues of the mice in the model, cisplatin, and GS-CDsgroup. Data were mean ± s.d. (n = 4). *p < 0.05, **p < 0.01 and ***p < 0.001 relative to the model group or cisplatin group, as analyzed by one-way ANOVA.

Meanwhile, statistical diagrams for weight changes of mice in the control, control + GS-CDs, model, cisplatin, and GS-CDs group were also recorded to assess the toxicity of GS-CDs (Fig. 5e). Compared to the weight of mice in control group, weight of mice in control + GS-CDs group was slightly lower at the initial stage. With more time, weight of mice in control + GS-CDs group exceeded that of control group. This proved that the prepared GS-CDs had almost no toxic effects on organism. Compared to the weight of mice in model group, weight of mice in GS-CDs group was very significant. This also showed that GS-CDs did not produce toxic effects during neuroblastoma treatment. Attractively, because it could effectively inhibit tumor growth and improve health of mice, the weight of mice in GS-CDs group was close to the control group on the 13th day. The weight loss of mice in the cisplatin group was the most. The H&E staining of the main organs of mice in each group results showed that few obvious organ abnormalities were observed in the pathological images of mice in cisplatin group (Fig. 6a). Experimental observations found that the mice injected with cisplatin were in a depressed state compared to mice in other groups. They were reluctant to eat for a period of time. This was possibly due to the severe irritation of cisplatin on the intestines and stomachs of the mice, resulting in significant weight loss. The above results indicated that the GS-CDs had high biocompatibility in the treatment process and could be used safely and effectively to treat neuroblastoma. It should be emphasized that human neuroblastoma is mainly harmful to infants and young children with low immunity. For those children who need to be carefully protected during treatment, the side effects caused by the nano-drug GS-CDs based on natural herbal extracts would be greatly reduced. Thus, the acceptance rates for infants and young children would be much higher. It would be more conducive to the treatment of the disease.

To estimate pathological damage of GS-CDs on tumors, the dissected neuroblastoma of mice in the model, cisplatin, GS-CDs group were stained and analyzed (Fig. 6b-d). Because human neuroblastoma tumor cells were malignant and grew quickly, sizes of tumors in the control group were very large at the end of the test period. The provided blood and nutrients were insufficient for the tumor, causing necrosis of the cells inside tumor. There was abundant blood supply outside tumor, and the tumor could maintain rapid growth. As observed in the H&E (hematoxylin-eosin staining) image, tumor cells in each group had a certain degree of damage, including in the model group. Neovascularization is a necessary condition for tumor growth, and antigen CD31 (endothelial cell adhesion molecule) was employed to label the vascular endothelial cells in tumor. The expression of CD31 in GS-CDs group was clearly lower than the other two groups. In addition, antigen neuron-specific enolase (NSE) is a marker of neuroblastoma. Compared to the other groups, expression level of NSE in GS-CDs group was the lowest.

It was worthy to note that since we did not modify the surfaces of GS-CDs with brilliant commercial fluorescent agent or contrast agent, it was hard to study the exact biodistribution of GS-CDs in animals. We have checked the literatures within last ten years to speculate the fates of GS-CDs in animals (Additional file 1: Table S6-1) [16, 44–48].

## Conclusion

This study used GS of natural bioactivity as a single reactant to synthesize GS-CDs via a one-step hydrothermal method. The sizes of GS-CDs were concentrated in the range of 2–4 nm, with good water solubility. The optimal excitation and emission wavelengths were observed at ~ 360 and ~ 440 nm, respectively. At a low concentration of GS-CDs (50 µg·mL^− 1^) for 48 h, the cell inhibition rate reached ~ 45.00% and apoptosis rate of SH-SY5Y cells reached ~ 59.66%. GS-CDs prepared with GS as the only reactive carbon source had little cell toxicity and the optimal inhibition effect of SH-SY5Y cells. GS-CDs induced G2/M arrest of SH-SY5Y cell cycle. Western-blot experiments further confirmed the apoptosis of SH-SY5Y cells induced by GS-CDs may be through inhibiting the up-regulation of Bcl-2, activating the caspase apoptosis pathway, activating and up-regulating the expression of apoptosis-related proteins caspase-3 and caspase-10. In in vivo experiments, compared to the model and cisplatin groups, mice in GS-CDs group had the slowest tumor growth rate and the smallest tumor volume. The GS-CDs also showed benign response characteristics, such as normal weight and lively actions in the mice after frequent interventions. These low-cost GS-CDs, which had a simple preparation process and good curative effect, showed excellent application prospects for the clinical treatment of human neuroblastoma in vulnerable children.

All the above results suggested that the GS-CDs prepared with natural active substances as carbon sources retained a certain amount of medicinal activity. We speculated that the main GS structure was not completely destroyed during the self-assembly process to form the GS-CDs. Although some functional groups changed during the reaction, they also resulted in more active sites on the surface of the prepared CDs. These abundant functional groups were conducive to increasing the cell uptake rate and increasing the interactions between the nano-drugs and the cells. This possibly compensated for the decrease in medicinal properties due to the changes in functional groups of the GS, to some extent. Therefore, the reaction conditions, including the time, temperature, and concentration, had a relatively large impact on the structure of the CDs nanomedicine and the corresponding biological activity. Tracking the change relationship between the CDs structure and their medicinal activity during the preparation process will also be a topic that requires future attention and research. In summary, the GS-CDs prepared with natural active substances showed good medicinal effects and low biological toxicity. We hope to construct a new research direction focused on low-toxicity biopharmaceuticals, based on bioactive CDs which are constructed by natural medicinal molecules. This maybe become a key research direction for new nanomedicines in the future.

## Electronic supplementary material

Below is the link to the electronic supplementary material.

**Additional file 1**: Supplementary table and figures.

## Data Availability

Data will be made available on request.

## Abbreviations

CDs: Carbon nanodots
GS-CDs: Ginsenosides carbon nanodots
Glu-CDs: Glucose carbon nanodots
TEM: Transmission electron microscopy
HRTEM: High-resolution transmission electron microscopy
DMEM: Dulbecco’s modified eagle medium
DMEM/F-12: Dulbecco’s modified eagle medium/nutrient mixture F-12
MEM/EBSS: Eagle’s minimum essential medium with Earle’s balanced salts
RPMI-1640: Roswell park memorial institute medium
FBS: Fetal bovine serum
ECL: Enhanced chemiluminescence
DAPI: 4’6-diamidino-2-phenylindole dihydrochloride
OD: Optical density
XPS: X-ray photoelectron spectroscopy
SDS-PAGE: Sodium dodecyl sulphate-polyacrylamide gel
CCK-8: Cell counting kit-8

## References

[1] Rennick JJ, Johnston APR, Parton RG (2021). Key principles and methods for studying the endocytosis of biological and nanoparticle therapeutics. Nat Nanotechnol.

[2] Cortez-Jugo C, Czuba-Wojnilowicz E, Tan A, Caruso F (2021). A focus on “Bio” in bio-nanoscience: the impact of biological factors on nanomaterial interactions. Adv Healthc Mater.

[3] Jiang W, Kim BY, Rutka JT, Chan WC (2008). Nanoparticle-mediated cellular response is size-dependent. Nat Nanotechnol.

[4] Adams D, Gonzalez-Duarte A, O’Riordan WD, Yang C, Ueda M, Kristen AV, et al. O.B. Suhr, Patisiran, an RNAi therapeutic, for hereditary transthyretin amyloidosis, N Engl J Med. 2018;379:11–21. 10.1056/NEJMoa1716153 29972753

[5] Barenholz Y (2012). Doxil^®^—the first FDA-approved nano-drug: lessons learned. J Control Release.

[6] Sousa de Almeida M, Susnik E, Drasler B, Taladriz-Blanco P, Petri-Fink A. B. Rothen-Rutishauser, Understanding nanoparticle endocytosis to improve targeting strategies in nanomedicine, Chem Soc Rev. 2021;50:5397–5434. 10.1039/d0cs01127d PMC8111542 33666625

[7] Alemayehu YA, Ilhami FB, Manayia AH, Cheng C (2021). Mercury-containing supramolecular micelles with highly sensitive pH-responsiveness for selective cancer therapy. Acta Biomater.

[8] Pan X, Xu D, Tang X, Liu N, You Y, Wang X, Yan X, Ma X, Chen X (2020). Endocytosis-enabled construction of silica nanochannels crossing living cell membrane for transmembrane drug transport. Adv Funct Mater.

[9] Chen P, Lai J, Huang C (2021). Bio-inspired amphoteric polymer for triggered-release drug delivery on breast cancer cells based on metal coordination. ACS Appl Mater Inter.

[10] Zhou B, Wu Q, Hoover MWangA, Wang X, Zhou F, Towner RA, Smith N, Saunders D, Song J, Qu J, Chen WR (2020). Immunologically modified MnFe_2_O_4_ nanoparticles to synergize photothermal therapy and immunotherapy for cancer treatment. Chem Eng J.

[11] Cui X, Xu S, Wang X, Chen C (2018). The nano-bio interaction and biomedical applications of carbon nanomaterials. Carbon.

[12] Li S, Li L, Tu H, Zhang H, Silvester DS, Banks CE, Zou G, Hou H, Ji X (2021). The development of carbon dots: from the perspective of materials chemistry. Mater Today.

[13] Luo W, Zhang L, Yang Z, Guo X, Wu Y, Zhang W, Luo J, Tang T, Wang Y (2021). Herbal medicine derived carbon dots: synthesis and applications in therapeutics, bioimaging and sensing. J Nanobiotechnol.

[14] Yang ZC, Wang M, Yong AM, Wong SY, Zhang XH, Tan H, Chang AY, Li X, Wang J (2011). Intrinsically fluorescent carbon dots with tunable emission derived from hydrothermal treatment of glucose in the presence of monopotassium phosphate. Chem Commun.

[15] Truskewycz A, Yin H, Halberg N, Lai DTH, Ball AS, Truong VK, Rybicka AM, Cole I (2022). Therapeutic platforms: administration, distribution, metabolism, excretion, toxicity, and therapeutic potential. Small.

[16] Liu J, Geng Y, Li D, Yao H, Huo Z, Li Y, Zhang K, Zhu S, Wei H, Xu W, Jiang J, Yang B (2021). Deep red emissive carbonized polymer dots with unprecedented narrow full width at half maximum. Adv Mater.

[17] Liu X, Liu Y, Thakor AS, Kevadiya BD, Cheng J, Chen M, Li Y, Xu Q, Wu Q, Wu Y, Zhang G (2021). Endogenous NO-releasing Carbon Nanodots for Tumor-specific gas therapy. Acta Biomater.

[18] Du F, Zhang L, Zhang L, Zhang M, Gong A, Tan Y, Miao J, Gong Y, Sun M, Ju H, Wu C, Zou S (2017). Engineered gadolinium-doped carbon dots for magnetic resonance imaging-guided radiotherapy of tumors. Biomaterials.

[19] Bai Y, Zhao J, Zhang L, Wang S, Hua J, Zhao S, Liang H (2022). A smart near-infrared carbon dot-metal organic framework assemblies for tumor microenvironment-activated cancer imaging and chemodynamic-photothermal combined therapy. Adv Healthc Mater.

[20] Geng B, Xu S, Li P, Li X, Fang F, Pan D, Shen L (2022). Platinum crosslinked carbon dot@TiO_2 – x_ p-n junctions for relapse-free sonodynamic tumor eradication via high-yield ROS and GSH depletion. Small.

[21] Tong T, Hu H, Zhou J, Deng S, Zhang X, Tang W, Fang L, Xiao S, Liang J (2020). Glycyrrhizic-acid-based carbon dots with high antiviral activity by multisite inhibition mechanisms. Small.

[22] Liu J, Lu S, Tang Q, Zhang K, Yu W, Sun H, Yang B (2017). One-step hydrothermal synthesis of photoluminescent carbon nanodots with selective antibacterial activity against Porphyromonas gingivalis. Nanoscale.

[23] Li P, Liu S, Gao W, Zhang G, Yang X, Gong X, Xing X (2020). Low-toxicity carbon quantum dots derived from gentamicin sulfate to combat antibiotic resistance and eradicate mature biofilms. Chem Commun.

[24] Lin C, Chang L, Chu H, Lin H, Chang P, Wang PYL, Unnikrishnan B, Mao J, Chen S, Huang C (2019). High amplification of the antiviral activity of curcumin through transformation into carbon quantum dots. Small.

[25] Lu F, Ma Y, Huang H, Zhang Y, Kong H, Zhao Y, Qu H, Wang Q, Liu Y, Kang Z (2021). Edible and highly biocompatible nanodots from natural plants for the treatment of stress gastric ulcers. Nanoscale.

[26] Zhang M, Cheng J, Hu J, Luo J, Zhang Y, Lu F, Kong H, Qu H, Zhao Y (2021). Green Phellodendri Chinensis cortex-based carbon dots for ameliorating imiquimod-induced psoriasis-like inflammation in mice. J Nanobiotechnol.

[27] Teixidor-Toneu I, Jordan FM, Hawkins JA (2018). Comparative phylogenetic methods and the cultural evolution of medicinal plant use. Nat Plants.

[28] Xiong X, Guo J, Liao Q, Li Y, Zhou Q, Bi G, Li C, Du R, Wang X, Sun T, Guo L, Liang H, Lu P, Wu Y, Zhang Z, Ro D, Shang Y, Huang S, Yan J (2021). The Taxus genome provides insights into paclitaxel biosynthesis. Nat Plants.

[29] Chopra P, Chhillar H, Kim YJ, Jo IH, Kim ST, Gupta R. Phytochemistry of ginsenosides: recent advancements and emerging roles, Crit Rev Food Sci Nutr. 2021;1–28. 10.1080/10408398.2021.1952159 34278879

[30] Ratan ZA, Haidere MF, Hong YH, Park SH, Lee JO, Lee JS, Cho JY (2021). Pharmacological potential of ginseng and its major component ginsenosides. J Ginseng Res.

[31] Ahuja A, Kim JH, Kim JH, Yi YS, Cho JY (2018). Functional role of ginseng-derived compounds in cancer. J Ginseng Res.

[32] Park JA, Cheung NKV (2020). Targets and antibody formats for immunotherapy of neuroblastoma. J Clin Oncol.

[33] Louis CU, Shohet JM (2015). Neuroblastoma: molecular pathogenesis and therapy. Annu Rev Med.

[34] Wu S, Li W, Zhou W, Zhan Y, Hu C, Zhuang J, Zhang H, Zhang X, Lei B, Liu Y (2018). Large-scale one-step synthesis of carbon dots from yeast extract powder and construction of carbon dots/PVA fluorescent shape memory material. Adv Opt Mater.

[35] Sung CK, Hong KA, Lin S, Lee Y, Cha J, Lee JK, Hong CP, Han BS, Jung SI, Kim SH, Yoon KS (2009). Dual-modal nanoprobes for imaging of mesenchymal stem cell transplant by MRI and fluorescence imaging. Korean J Radiol.

[36] Brizzolara A, Garbati P, Vella S, Calderoni M, Quattrone A, Tonini GP, Capasso M, Longo L, Barbieri R, Florio T, Pagano A (2020). Co-administration of Fendiline Hydrochloride enhances chemotherapeutic efficacy of cisplatin in Neuroblastoma Treatment. Molecules.

[37] Peng H, Jin H, Zhuo H, Huang H (2017). Enhanced antitumor efficacy of cisplatin for treating ovarian cancer in vitro and in vivo via transferrin binding. Oncotarget.

[38] Zhu S, Song Y, Shao J, Zhao X, Yang B (2015). Non-conjugated polymer dots with crosslink-enhanced emission in the absence of fluorophore units. Angew Chem Int Edit.

[39] Zhu S, Zhang J, Wang L, Song Y, Zhang G, Wang H, Yang B (2012). A general route to make non-conjugated linear polymers luminescent. Chem Commun.

[40] Gu ZG, Li DJ, Zheng C, Kang Y, Wçll C, Zhang J (2017). MOF-templated synthesis of ultrasmall photoluminescent carbon- nanodot arrays for optical applications. Angew Chem Int Ed.

[41] Tang L, Ji R, Cao X, Lin J, Jiang H, Li X, Teng KS, Luk CM, Zeng S, Hao J, Lau SP (2012). Deep ultraviolet photoluminescence of water-soluble self-passivated graphene quantum dots. ACS Nano.

[42] Edlich F (2018). BCL-2 proteins and apoptosis: recent insights and unknowns. Biochem Biophys Res Commun.

[43] COHEN GM (1997). Caspases: the executioners of apoptosis. Biochem J.

[44] Yu N, Huang T, Duan T, Bao Y, Gao R, Wang X, Xu K, Han C (2022). Accurate detection and delineation boundary of renal cell carcinoma based on dual-targeted magnetic-fluorescent carbon dots. Chem Eng J.

[45] Ji DK, Dali H, Guo S, Malaganahally S, Vollaire J, Josserand V, Dumortier H, Ménard-Moyon C, Bianco A (2022). Multifunctional Carbon Nanodots: enhanced Near-Infrared Photosensitizing, Photothermal Activity, and body clearance. Small Sci.

[46] Liao J, Yao Y, Lee CH, Wu Y, Li P (2021). Vivo Biodistribution, Clearance, and Biocompatibility of multiple Carbon Dots containing nanoparticles for Biomedical Application. Pharmaceutics.

[47] Bao X, Yuan Y, Chen J, Zhang B, Li D, Zhou D, Jing P, Xu G, Wang Y, Holá K, Shen D, Wu C, Song L, Liu C, Zbořil R, Qu S (2018). In vivo theranostics with near-infrared-emitting carbon dots—highly efficient photothermal therapy based on passive targeting after intravenous administration. Light-Sci Appl.

[48] Huang X, Zhang F, Zhu L, Choi KY, Guo N, Guo J, Tackett K, Anilkumar P, Liu G, Quan Q, Choi HS, Niu G, Sun YP, Lee S, Chen X (2013). Effect of injection routes on the biodistribution, clearance, and tumor uptake of carbon dots. ACS Nano.

