# Supplementary material for: Carbon nanodots constructed by ginsenosides and their high inhibitory effect on neuroblastoma
Source: J Nanobiotechnology. 2023 Jul 28;21:244. doi: 10.1186/s12951-023-02023-w (PMC10386222; doi:10.1186/s12951-023-02023-w)
Supplement: Supplementary file 1 — Additional file 1: Supplementary table and figures. [file 12951_2023_2023_MOESM1_ESM.docx]

**Supplementary Information**

**Carbon nanodots constructed by ginsenosides and their high inhibitory effect on neuroblastoma**

Yingnan Jiang^1,†^, Lizhi Xiao^1,†^, Jifeng Wang^1^, Tenghui Tian^1^, Guancheng Liu^2^, Yu Zhao^1,*^, Jiajuan Guo^1^, Wei Zhang^1^, Jiawen Wang^1^, Changbao Chen^1,*^, Wenyi Gao^1,*^, Bai Yang^2^

^1^ Jilin Ginseng Academy, Changchun University of Chinese Medicine, Changchun, 130117, P. R. China

^2^ State Key Laboratory of Supramolecular Structure and Materials, College of Chemistry, Jilin University, Changchun, 130012, P. R. China

^†^These authors contributed to the work equally and should be regarded as co-first author.

^*^ These authors should be co-corresponding authors.

^*^Corresponding authors at: Jilin Ginseng Academy, Changchun University of Chinese Medicine, Changchun, 130117, P. R. China

E-mail address: cnzhaoyu1972@126.com (Y. Zhao); ccb2021@126.com (C. Chen); gaowy@ccucm.edn.cn (W. Gao).





**Fig. S1-1** UV spectrum of the GS aqueous solution before hydrothermal reaction.





**Fig. S1-2** The IR spectra of GS (orange line), GS-CDs@3h (red line), GS-CDs@5h (blue line), GS-CDs@6h (green line), and GS-CDs@10h (purple line), respectively.

In the IR spectra of the four GS-CDs, we observed absorption peaks at 3370, 2925, 2853, 1780–1550, 1458, 1386, 1075, and 1040 cm^−1^. These were attributed to the vibrations and rotations of O-H, C-H, C=O, and C-O.

Compared to the weak ν(C=O) in GS, obvious ν(C=O) was seen in the four GS-CDs. This was because -OH in GS underwent an oxidation reaction, forming C=O during the intense hydrothermal reaction.

Except for ν(C=O) absorption, almost all of the absorption peaks for GS-CDs@3h were stronger than the other GS-CDs. Because the heating time was short, many of the reaction sites on the tetracyclic triterpenes were in an active state. Thus, the longer the reaction time, the more buckle cross-links that formed *via* molecular self-assembly. Therefore, the ν(O-H) and ν(C-O) of GS-CDs@5h and GS-CDs@6h decreased slightly, compared to GS-CDs@3h.

In the infrared spectrum of GS-CDs@10h, except for ν(C=O), absorption was lower than the other three GS-CDs. Because the reaction time was increased to 10 h, the dissociation of the glycosyl group on the molecular branches was maximized. Therefore, the dissociation of the glycosyl branch caused the formed GS-CDs structure to be most rigid.

We inferred that the longer the reaction time, the higher the degree of crosslinking, causing the GS-CDs structure to become more decent and orderly, with enhanced UV absorption and fluorescence emissions (Fig. 1).


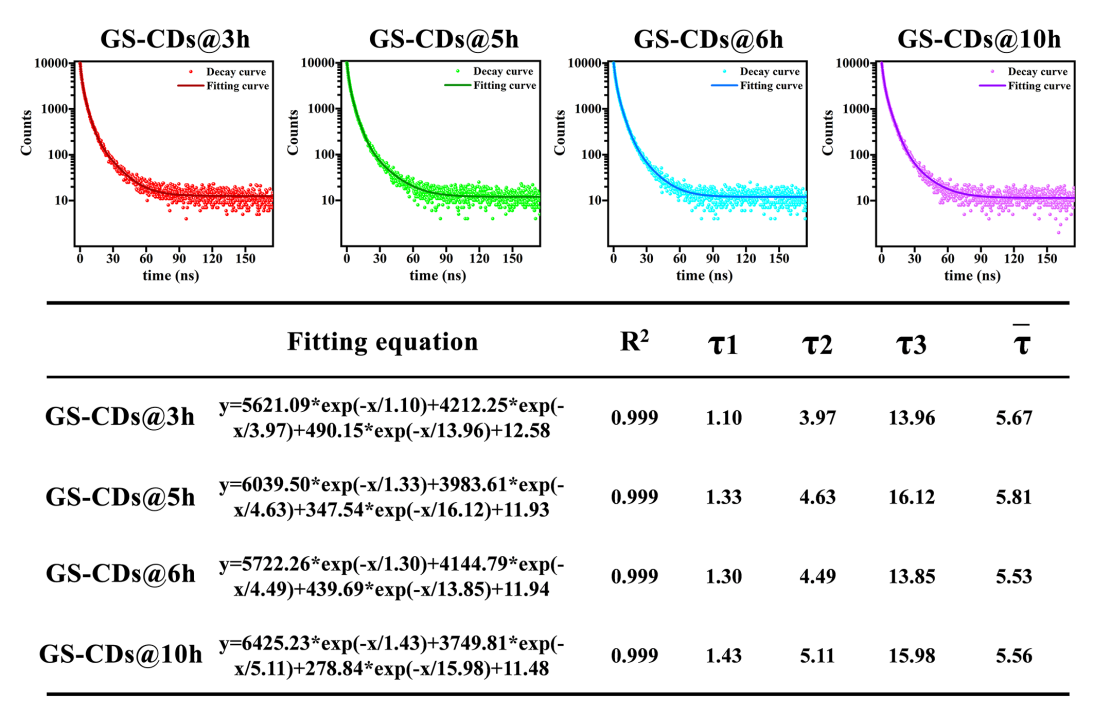


**Fig. S1-3** Fluorescence attenuation curves and fluorescence lifetimes of GS-CDs@3h, GS-CDs@5h, GS-CDs@6h, and GS-CDs@10h. The fluorescence attenuation curves of GS-CDs@3h (red), GS-CDs@5h (green), GS-CDs@6h (blue), and GS-CDs@10h (purple). After fitted with a third-order decay exponential function, three fluorescence lifetimes (τ) were obtained, as shown in the table below. The average fluorescence lifetimes of GS-CDs@3h, GS-CDs@5h, GS-CDs@6h, and GS-CDs@10h were 5.67, 5.81, 5.53, and 5.56 ns, respectively.


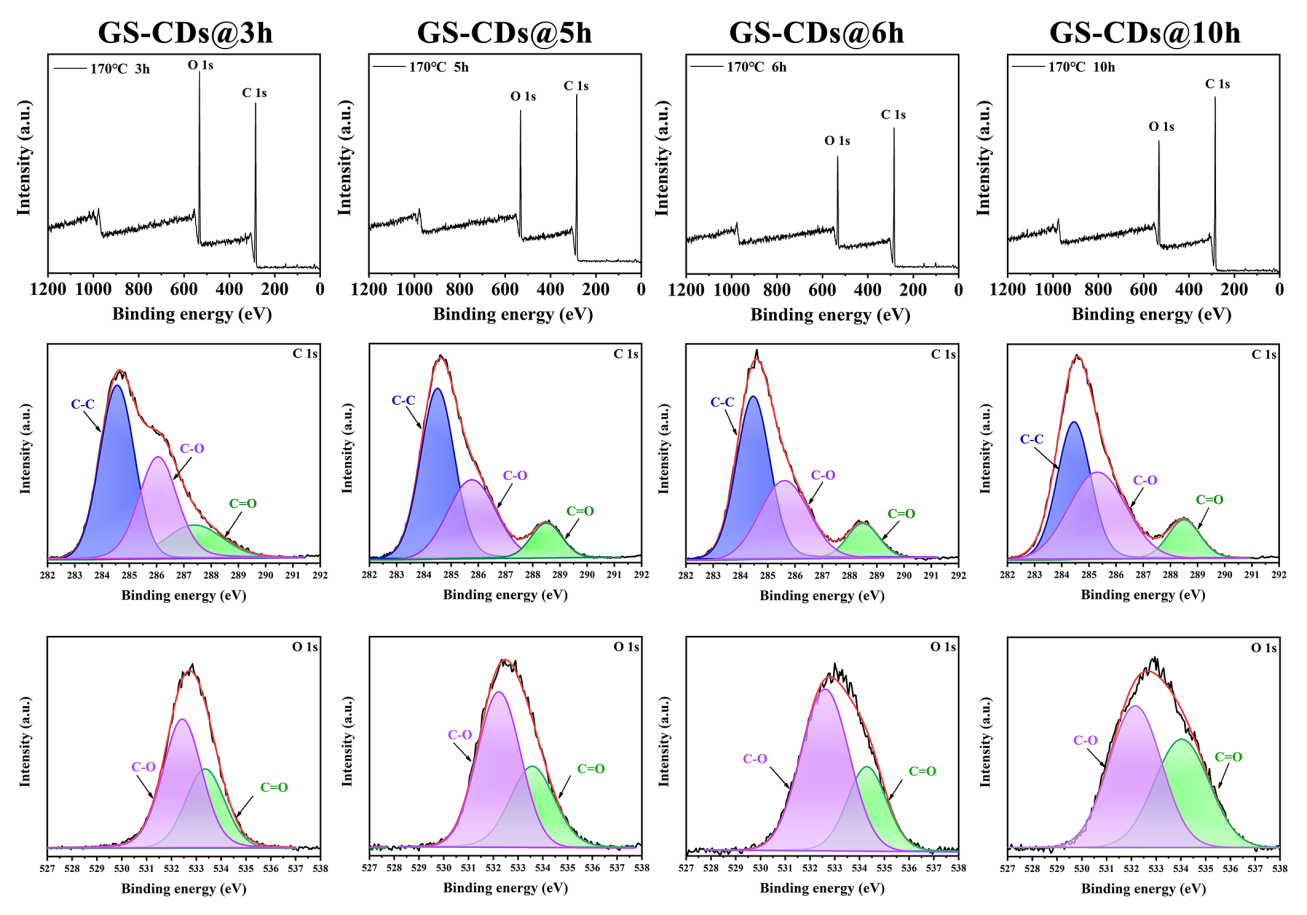


**Fig. S1-4** XPS spectra and peak splitting of GS-CDs@3h, GS-CDs@5h, GS-CDs@6h, and GS-CDs@10h.


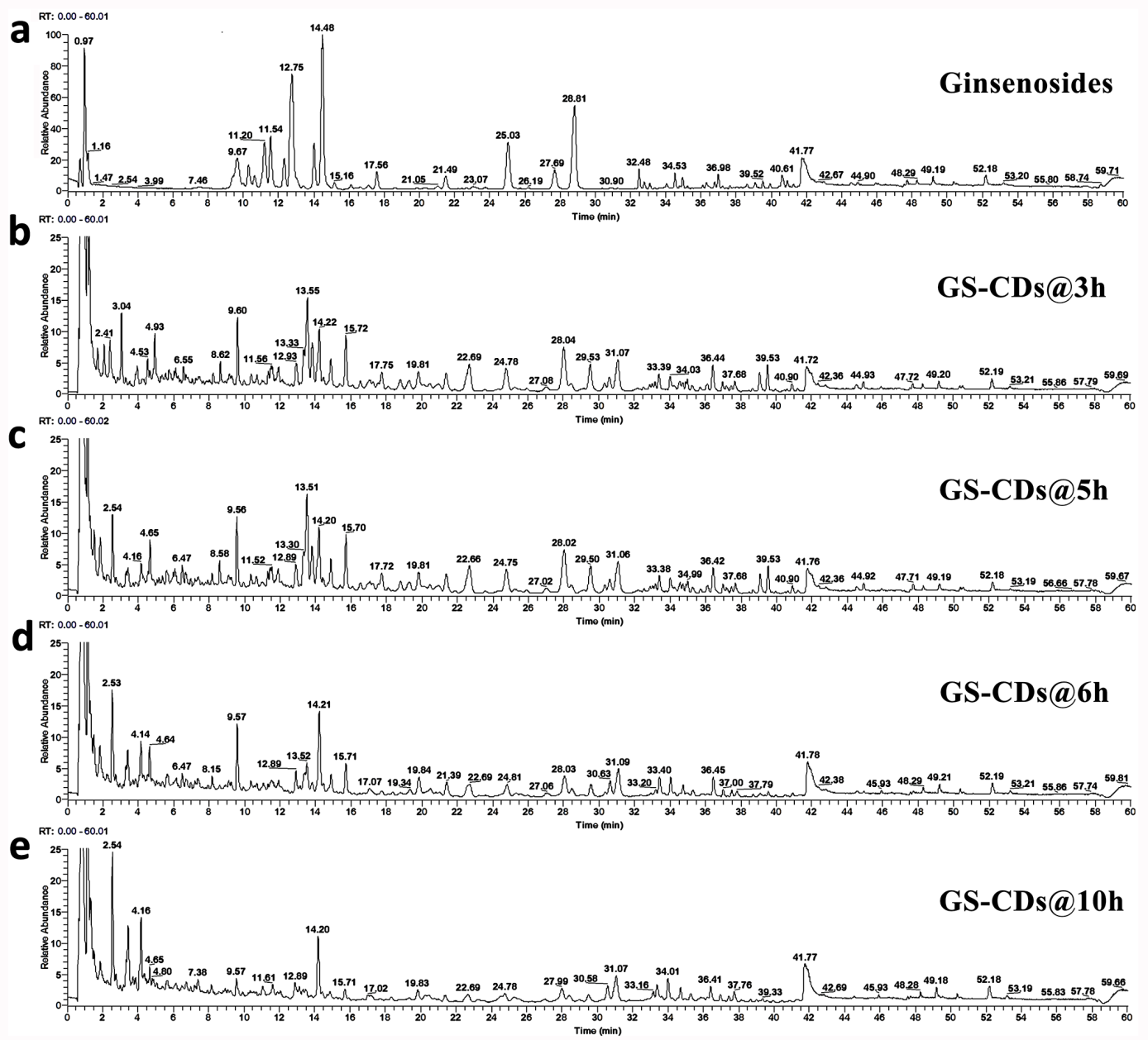


**Fig. S1-5** Total ion current chromatograms of GS (a), GS-CDs@3h (b), GS-CDs@5h (c), GS-CDs@6h (d) and GS-CDs@10h (e), respectively.

**Table S1-1.** Analysis results of mass spectra of GS，GS-CDs@3h, GS-CDs@5h, GS-CDs@6h, and GS-CDs@10h. The compounds and formula of detected ginsenosides have been depicted one by one.


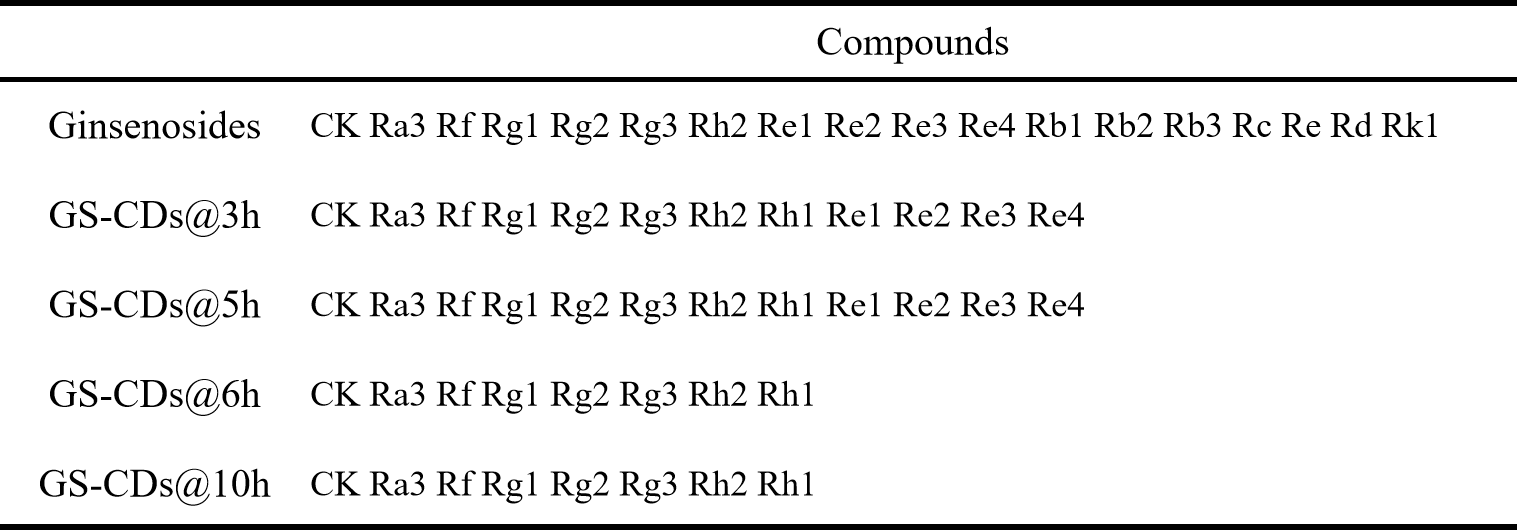


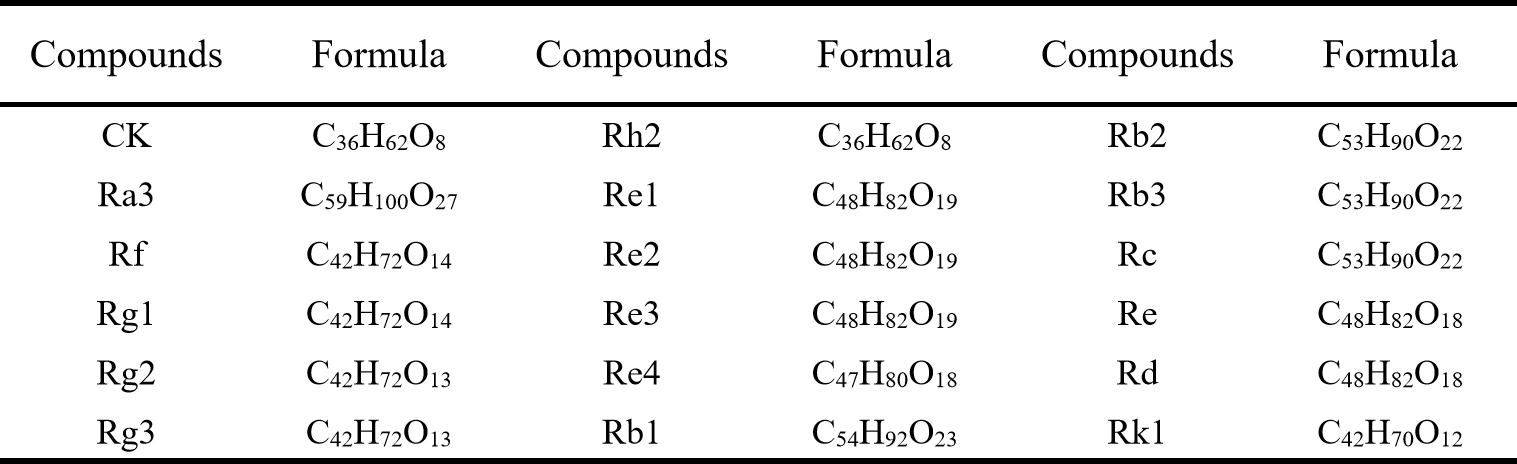


**
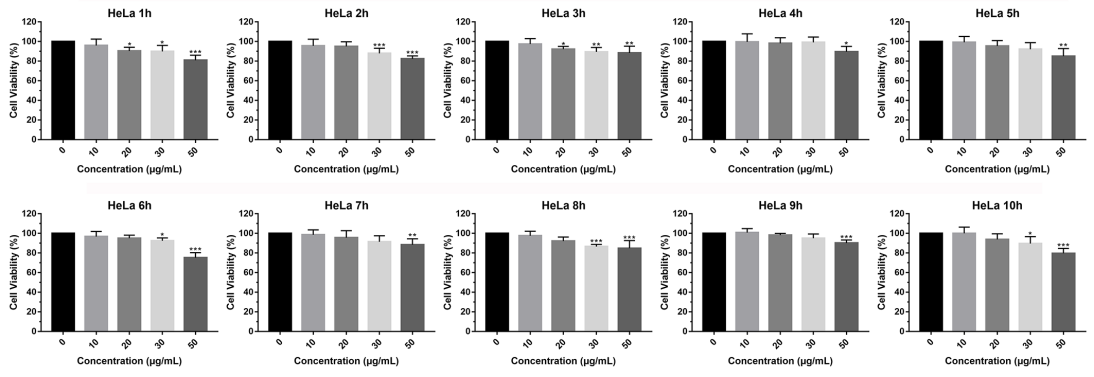
**

**Fig. S2-1** *In vitro* cytotoxicity profiles of GS-CDs@1h, GS-CDs@2h, GS-CDs@3h, GS-CDs@4h, GS-CDs@5h, GS-CDs@6h, GS-CDs@7h, GS-CDs@8h, GS-CDs@9h, and GS-CDs@10h on HeLa cells. Data are mean ± s.d. (*n*=6). **p*<0.05, ***p*<0.01 and ****p*<0.001 relative to 0 *μ*g mL^-1^, as analyzed by one-way ANOVA.

**
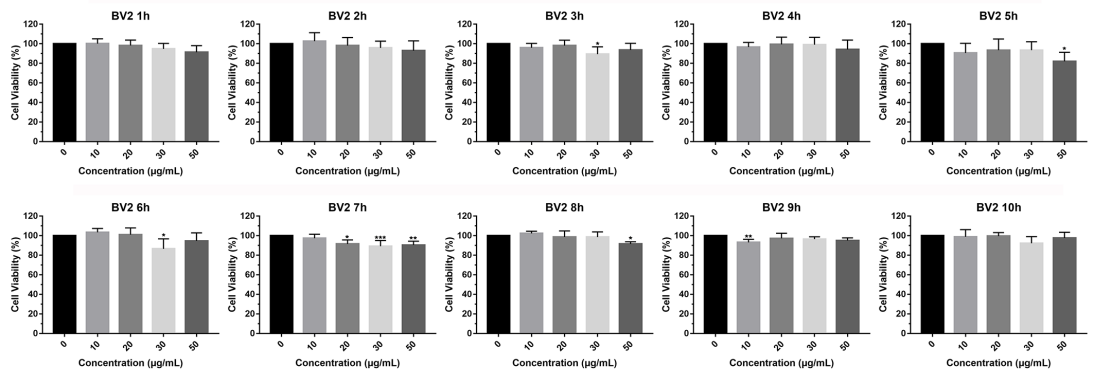
**

**Fig. S2-2.** *In vitro* cytotoxicity profiles of GS-CDs@1h, GS-CDs@2h, GS-CDs@3h, GS-CDs@4h, GS-CDs@5h, GS-CDs@6h, GS-CDs@7h, GS-CDs@8h, GS-CDs@9h, and GS-CDs@10h on BV2 cells. Data are mean ± s.d. (*n*=6). **p*<0.05, ***p*<0.01 and ****p*<0.001 relative to 0 *μ*g mL^-1^, as analyzed by one-way ANOVA.

**
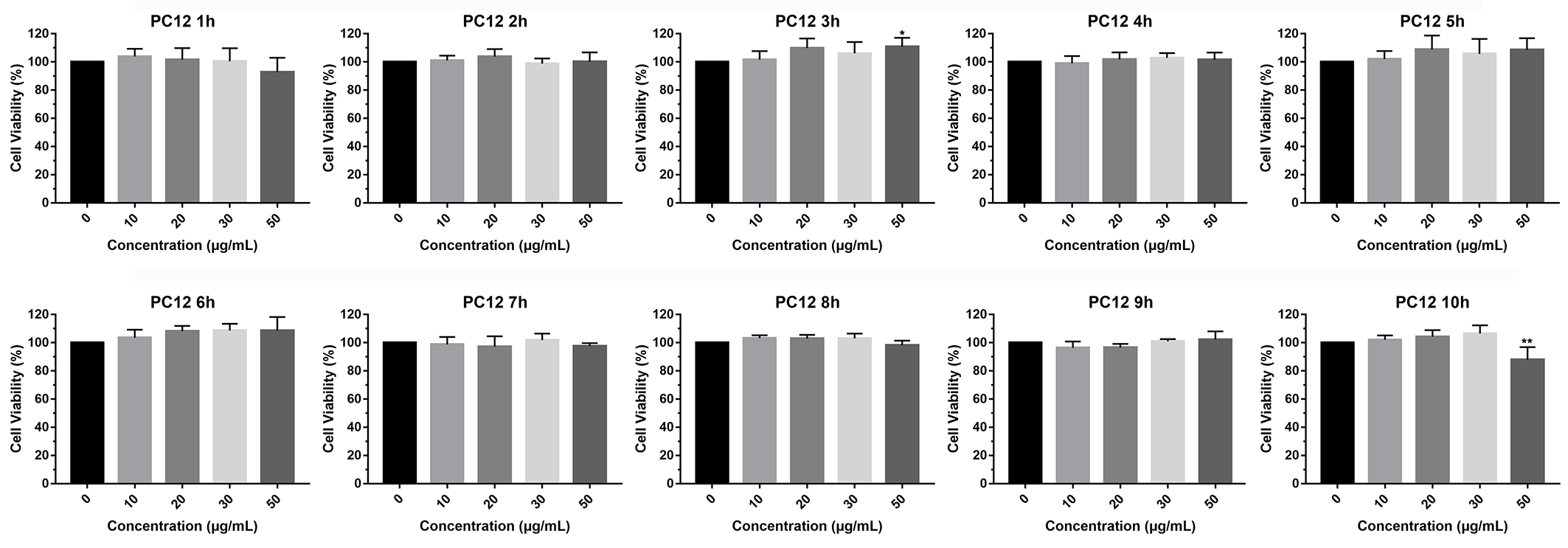
**

**Fig. S2-3.** *In vitro* cytotoxicity profiles of GS-CDs@1h, GS-CDs@2h, GS-CDs@3h, GS-CDs@4h, GS-CDs@5h, GS-CDs@6h, GS-CDs@7h, GS-CDs@8h, GS-CDs@9h, and GS-CDs@10h on PC12 cells. Data are mean ± s.d. (*n*=6). **p*<0.05, ***p*<0.01 and ****p*<0.001 relative to 0 *μ*g mL^-1^, as analyzed by one-way ANOVA.

**
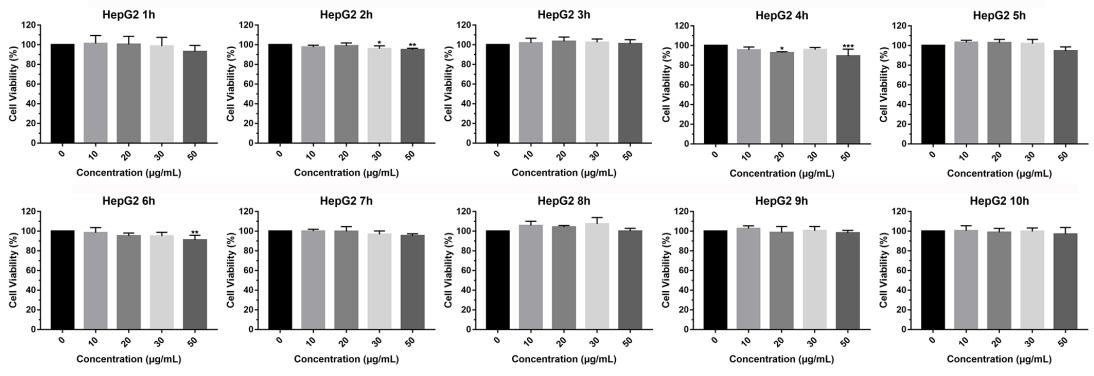
**

**Fig. S2-4.** *In vitro* cytotoxicity profiles of GS-CDs@1h, GS-CDs@2h, GS-CDs@3h, GS-CDs@4h, GS-CDs@5h, GS-CDs@6h, GS-CDs@7h, GS-CDs@8h, GS-CDs@9h, and GS-CDs@10h on HepG2 cells. Data are mean ± s.d. (*n*=6). **p*<0.05, ***p*<0.01 and ****p*<0.001 relative to 0 *μ*g mL^-1^, as analyzed by one-way ANOVA.

**
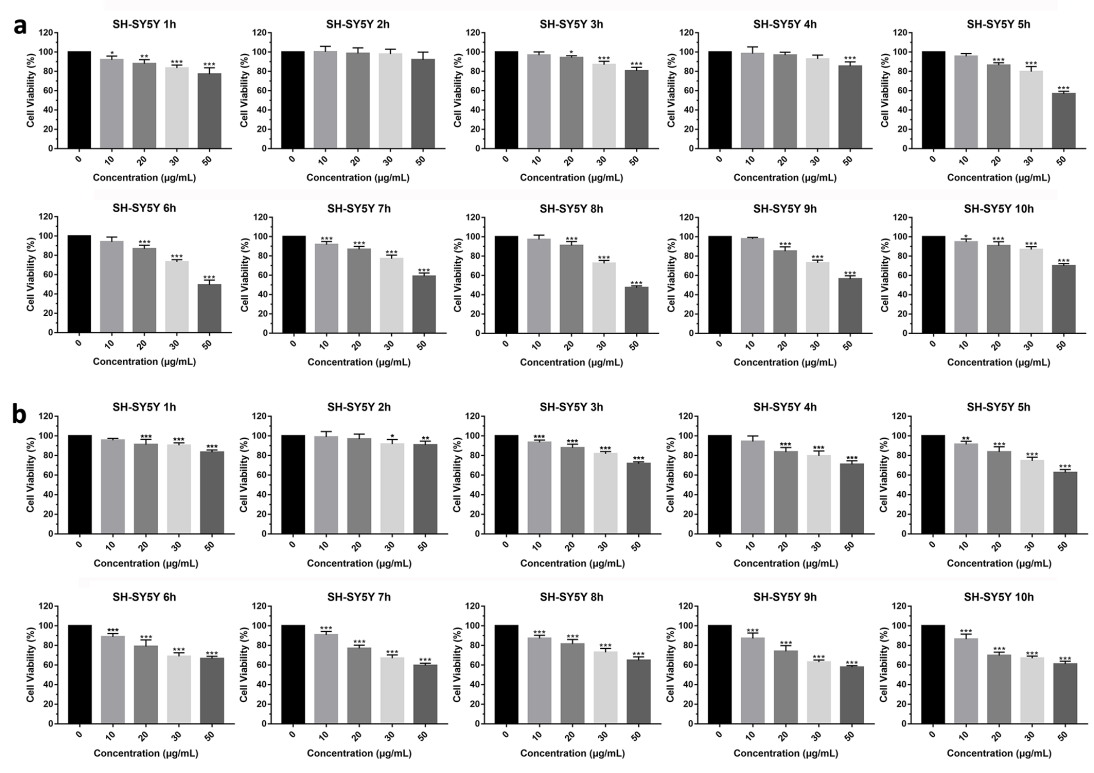
**

**Fig. S2-5.** Profiles showing two *in vitro* cytotoxicity iterations of GS-CDs@1h, GS-CDs@2h, GS-CDs@3h, GS-CDs@4h, GS-CDs@5h, GS-CDs@6h, GS-CDs@7h, GS-CDs@8h, GS-CDs@9h, and GS-CDs@10h on SH-SY5Y cells. Data are mean ± s.d. (*n*=6). **p*<0.05, ***p*<0.01 and ****p*<0.001 relative to 0 *μ*g mL^-1^, as analyzed by one-way ANOVA.

**
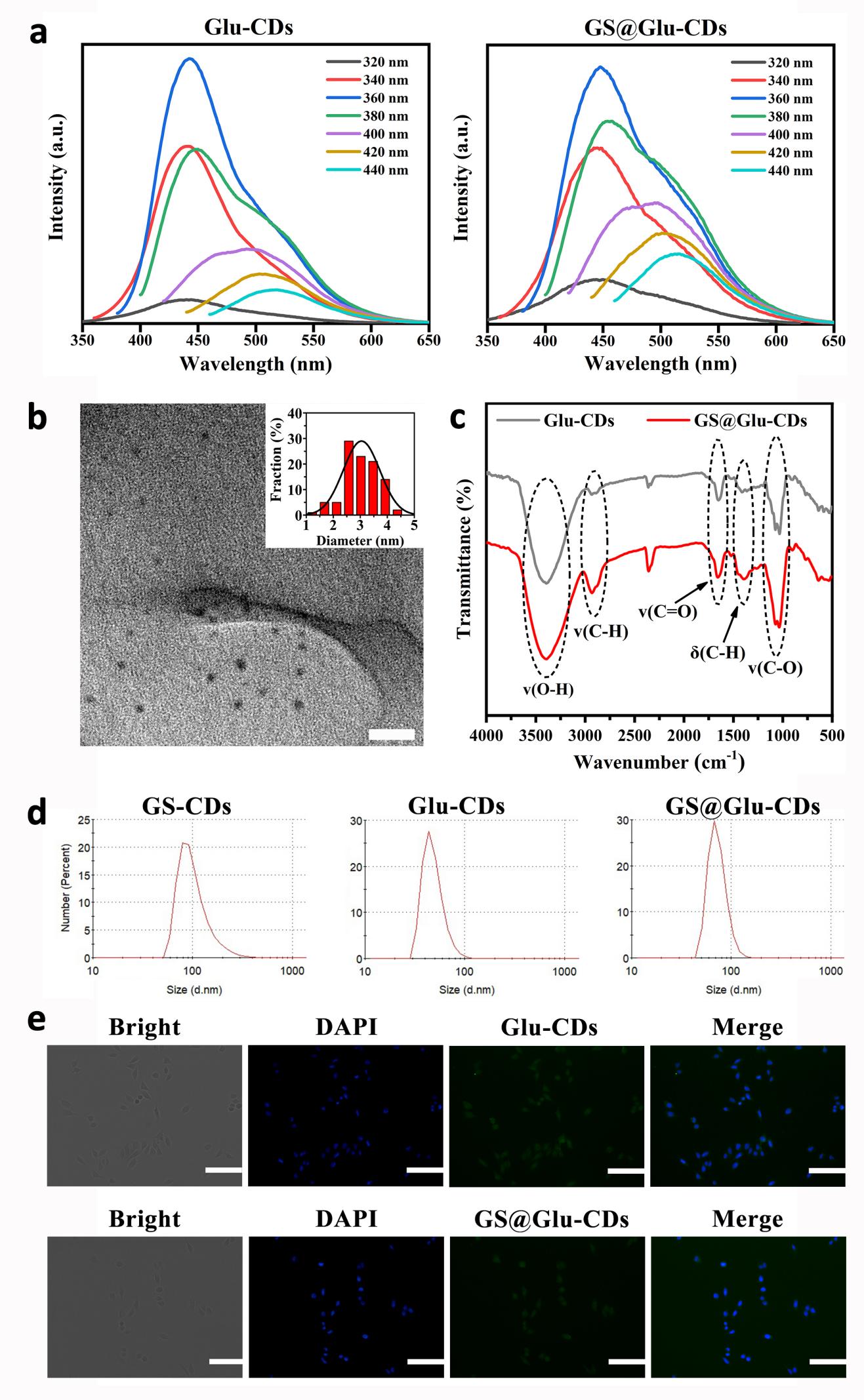
**

**Fig. S2-6** **a** Fluorescence spectra of Glu-CDs (left) and GS@Glu-CDs (right), showed excitation-dependent properties of the CDs. **b** TEM image of GS@Glu-CDs. The size distribution of GS@Glu-CDs was concentrated at ~ 3.20 ± 0.57 nm. The scale bar was 20 nm. **c**. FTIR spectra of Glu-CDs (gray line) and GS@Glu-CDs (red line). **d** DLS analysis of GS-CDs, Glu-CDs and GS@Glu-CDs. **e** Fluorescence microscope images of Glu-CDs (top) and GS@Glu-CDs (bottom) uptake in SH-SY5Y cells. At the same concentration of 100 *μ*g mL^-1^ and after 6 h of incubation, both Glu-CDs and GS@Glu-CDs entered SH-SY5Y cells, showing green fluorescence. The scale bars were 50 *μ*m.

In Fig. S2-6a, some differences between the excitation-dependent fluorescence spectra between the two CDs were observed. For example, at the same optimal excitation wavelength (360 nm), optimal emission wavelengths for Glu-CDs (left) and GS@Glu-CDs (right) were 442 and 448 nm, respectively. These differences were possibly caused by the loading of GS on the Glu-CDs, which affected the fluorescence properties.

In Fig. S2-6c, the FTIR spectrum of GS@Glu-CDs was similar to that of Glu-CDs. It implied that there were hardly new chemical bonds between GS and Glu-CDs. The interaction between GS and Glu-CDs might be supramolecular force.

As can be seen in Fig. S2-6d, the hydrodynamic radius distribution of GS-CDs was concentrated around ~ 78.82 nm, the hydrodynamic radius distribution of Glu-CDs was concentrated around ~ 43.83 nm, and the hydrodynamic radius distribution of GS@Glu-CDs was concentrated around ~ 68.06 nm. Note that the hydrodynamic radius distribution of GS@Glu-CDs was a little larger than that of Glu-CDs. It was further confirmed that the interaction between GS and Glu-CDs was supramolecular force.

All these analysis in Fig. S2-6 proved that GS@Glu-CDs has been successfully obtained.

**
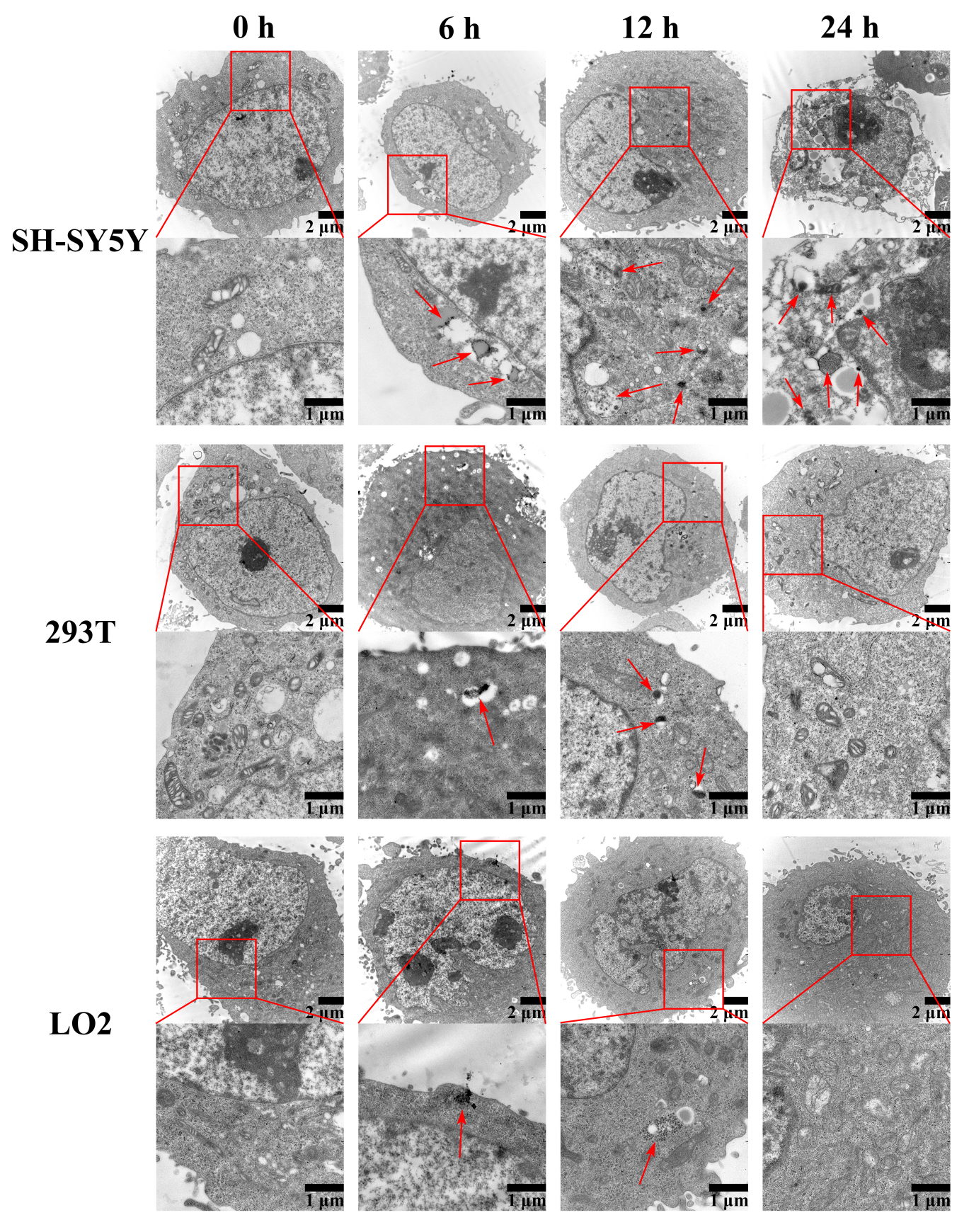
**

**Fig. S3-1** Biological section TEM images of coculture of GS-CDs with SH-SY5Y, 293T and LO2 cells at 0 6 12 and 24 h, respectively.

**
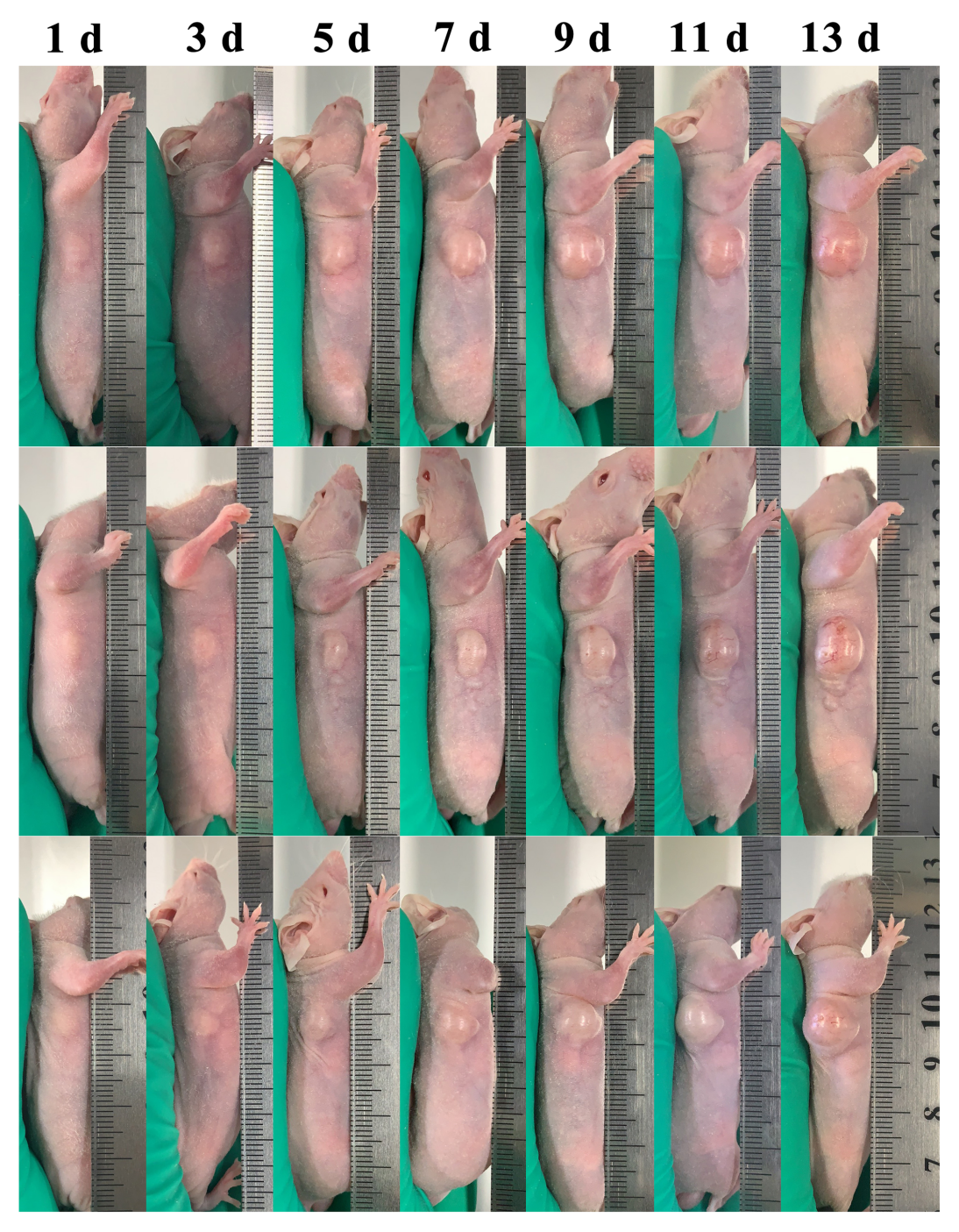
**

**Fig. S5-1** Real-time photos of mouse growth every 2 d in the model group.

**
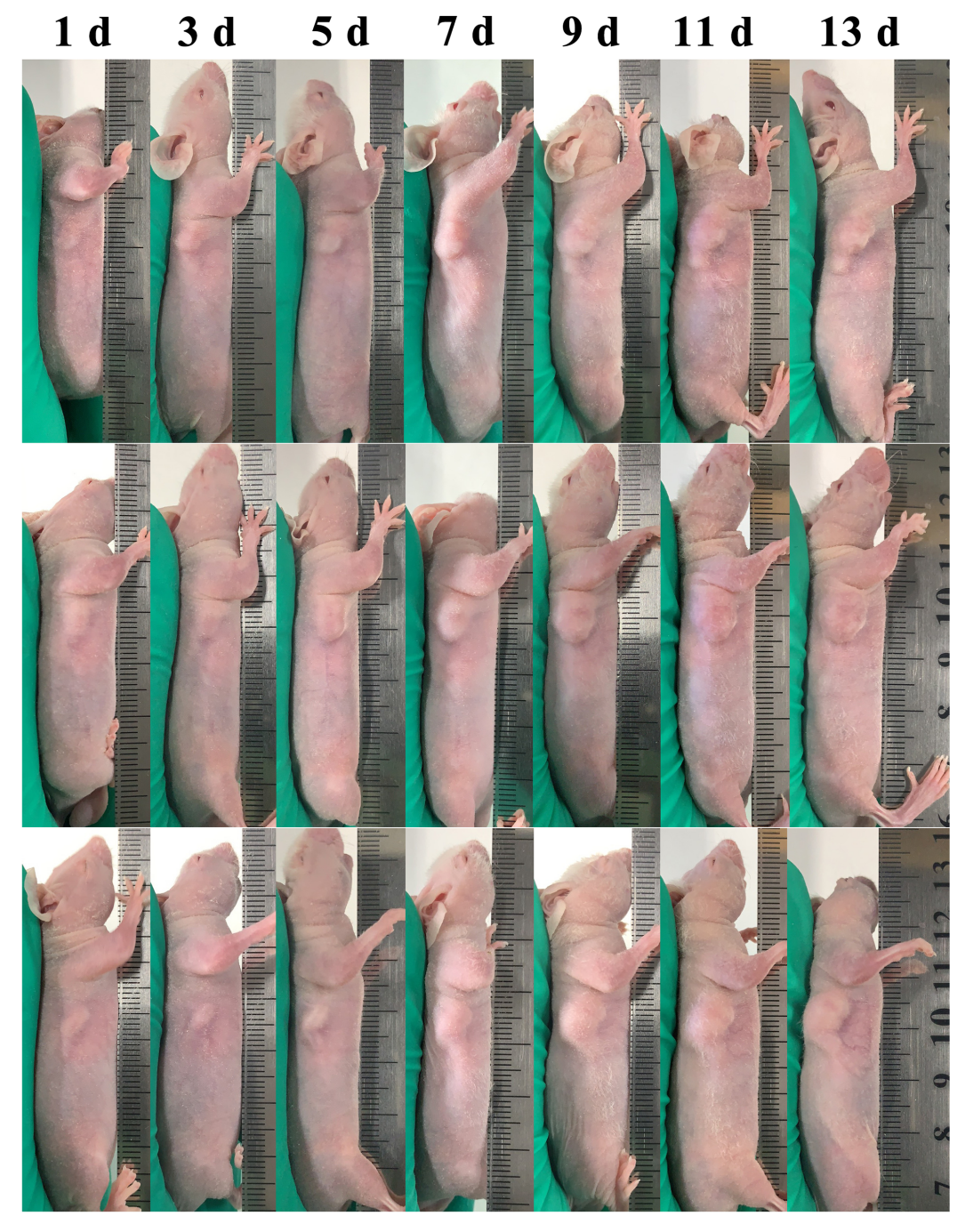
**

**Fig. S5-2** Real-time photos of mouse growth every 2 d in the cisplatin group.

**
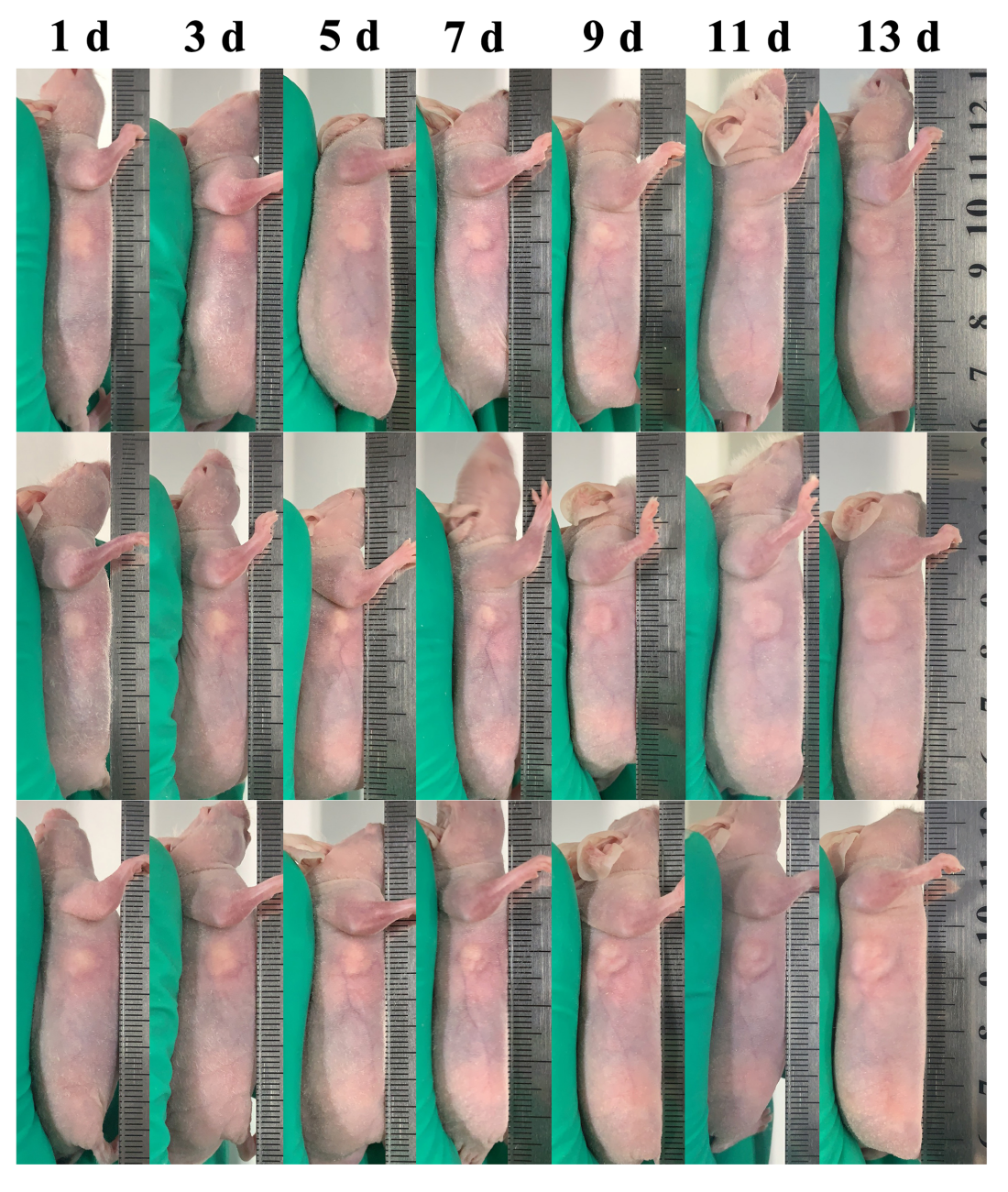
**

**Fig. S5-3** Real-time photos of mouse growth every 2 d in the GS-CDs group.

| **Table S6-1** Previous literature surveys on biodistribution and clearance of multiple CDs in vivo. | | | | | | | |  |
| --- | --- | --- | --- | --- | --- | --- | --- | --- |
| **Reference** | **^a^Label** | **Material** | **Size** | **hydrodynamic radius** | **Surface functional group** | **Biodistribution** | **Clearance** | |
| 16 | CPDs | Taxus leaves and acetone solution | 2.7 ± 0.7 nm | 529.8 ± 162.8 nm | amide and hydroxy functional groups | liver, lung, and kidney | Excreted via the kidney system (1~2 h) and hepatobiliary system (24 h). | |
| 44 | AS1411-CLT1-Gd-CDs | Magnevist (Gd-DTPA), L-arginine (L-Arg), AS1411 aptamer and CLT1 peptide | n/a | 7.63 nm | amino, carboxylate, and hydroxy functional groups | tumor, kidney, ureter and urinary bladder | Cleared by urinary system within 12 h. | |
| 45 | MCNDs | polythiophene phenylpropionic acid, folic acid and chlorin e6 (Ce6) | ~5 nm | 40-90 nm | amide and carboxyl functional groups | liver, kidney, intestines | hepatobiliary and renal elimination (>24 h) | |
| 46 | CDs@PEI | methyl methacrylate and polyethyleneimine (PEI) | 99.5 nm | 109 nm | nonionic methoxy poly(ethylene glycol) groups | tumor, liver, kidney | Hepatobiliary excretion (30 h) | |
| 47 | NIR CDs | citric acid, urea and DMSO | 2~5 nm | n/a | carboxyl and carbonyl functional groups | tumor, kidney, liver | Excreted via the kidney system (24 h) | |
| 48 | C-dots | C-dots, diamineterminated oligomeric poly(ethylene glycol), ZW800 | ~3 nm | n/a | hydroxy and carboxyl functional groups | tumor, kidney | Excreted via the kidney system (24 h) | |
| ^a^ Labels indicate either additional details regarding the nature of the reported carbon nanodot or indicate the abbreviation/common label used within the cited study to describe the particle. | | | | | | | |  |

It can be concluded from the previously reports, various CDs tended to be accumulated in liver, kidney and tumor tissues. In that case, we have studied the traces of GS-CDs in SH-SY5Y, LO2 and 293T cells by TEM from 0-24 h. Fig. S3-1 showed biological section TEM images of coculture of GS-CDs with SH-SY5Y, 293T and LO2 cells at 0 6 12 and 24 h, respectively. Many GS-CDs entered the SH-SY5Y cells and mainly existed in the cytoplasm. At 24 h, GS-CDs were still abundant in SH-SY5Y cells, that some cells shrank, some cell membranes were broken, and some cell shapes were destroyed. This indicated that GS-CDs can be accumulated in SH-SY5Y cells and inhibit effectively. Within 0-12h, GS-CDs also entered into 293T and LO2 cells. At 24h, few GS-CDs could be found in 293T and LO2 cells, indicating that GS-CDs were basically metabolized within 24h. And 293T and LO2 cells had normal morphology and good growth status at 24h. It proved that GS-CDs had no toxic effect on normal cells 293T and LO2.
